# Effects of cognitive and stress management training in middle-aged and older industrial workers in different socioeconomic settings: a randomized controlled study

**DOI:** 10.3389/fpsyg.2023.1229503

**Published:** 2023-09-12

**Authors:** Patrick D. Gajewski, Catharina Stahn, Joachim Zülch, Edmund Wascher, Stephan Getzmann, Michael Falkenstein

**Affiliations:** ^1^Leibniz Research Centre for Working Environment and Human Factors at TU Dortmund (IfADo), Dortmund, Germany; ^2^Ifaa – Institute of Applied Industrial Engineering and Ergonomics, Düsseldorf, Germany; ^3^Industrial Sales Engineering, Ruhr-Universität Bochum (RUB), Bochum, Germany; ^4^Institute for Working Learning Ageing (ALA), Bochum, Germany

**Keywords:** aging, aging workforce, industry workers, cognitive training, stress management, neuropsychology, cortisol awake response (CAR)

## Abstract

**Introduction:**

The demographic change requires longer working lifetime. However, fear of job loss may lead to chronic stress whereas aging and unchallenging work may accelerate cognitive decline and early retirement. Long-time repetitive work led to impairments of cognitive functions in middle-aged and older employees, as demonstrated in a previous study conducted in a large car manufacturer. In the present study, a training concept was implemented to enhance the cognitive and emotional competence of these employees.

**Methods:**

A first group of employees received a trainer-guided cognitive training only, whereas a wait list control group received a cognitive training and stress management training. This design was applied in two independent samples separated by one year either during or after a socioeconomically tense situation of the factory.

**Results:**

In sample 1, with a tense occupational situation, the cognitive training effects occurred with a delay of three months. In contrast, in sample 2, with less critical socioeconomic situation, the training effects occurred immediately and persisted three months later. Stress management training showed reduction of subjectively and objectively measured stress level.

**Discussion:**

The results indicate that effects of cognitive interventions are diminished under chronic stress which can be reduced after a short stress management training. This leads also to enhanced attention and memory in daily life. In contrast, in Sample 2 with less chronic stress, effects of cognitive training were stronger and persisted at least three months later, whereas stress management training had less impact. This suggests that cognitive learning in occupational settings is only efficient at lower stress levels.

## Introduction

The demographic change, that is, the decreasing ratio of young and increasing ratio of older people, will lead to longer working lifetime and delayed retirement. On the other hand, increasing workload, economic and financial crises, fear of job lost, and social relegation amplify the psychosocial job demands and, consequently, chronic stress level [Health-related quality of life (HRQOL), 2021]. Additionally, aging accompanied by chronic stress may lead to declines of cognitive functions crucial for daily life (Lupien et al., 2007, 2009).

Unimpaired cognitive functioning in aging, however, is crucial for both the occupational safety and health and productivity, since work activity requires the interplay of sensory, cognitive, motor, motivational, and emotional competences. While these functions arise and improve during adolescent development, they take on a very different course during adult life (Salthouse, 2010a,b). Some of these functions remain unchanged or even improve into older age, for example, general knowledge “crystalized functions” as well as linguistic and social competence (Lindenberger and Baltes, 1997; Grossmann et al., 2010; Salthouse, 2010b). Other (“fluid”) functions can already diminish from middle adulthood with individually different speed (Verhaeghen and Salthouse, 1997; Hedden and Gabrieli, 2004; Schroeder and Salthouse, 2004; Raz and Rodrigue, 2006; Salthouse, 2010b). Fluid functions that typically decline first are those which ensure fast and effective action control and goal-directed behavior under complex conditions, both in private and working life. These functions include short-term memory, working memory, action planning and preparation, orientation and control of attention, search for relevant information in the environment, inhibition of irrelevant information and reactions, multitasking, switching between tasks, and the monitoring of own actions such as detection and correction of errors (Schroeder and Salthouse, 2004; Raz and Rodrigue, 2006; Salthouse, 2010b; Diamond, 2013).

The development of fluid functions and their decline in older age are influenced less by calendar age than by individual lifestyle and environmental factors (Anstey et al., 2001). In terms of lifestyle factors, mental and physical activity keep older people cognitively fit (Gajewski and Falkenstein, 2011, 2012, 2016a,b, 2018; Ballesteros et al., 2015; Sedek et al., 2021; Strobach and Karbach, 2021). Moreover, mentally stimulating, or varied work is associated with maintenance of cognitive functions as occupation is recognized as a modifiable factor enhancing cognitive capacity in older adults, while long-term unchallenging, repetitive work with little room for flexibility lead to an accelerated decline in fluid cognitive functions (Schooler et al., 1999; Bosma et al., 2003; Andel et al., 2007, 2015; Marquie et al., 2010; Correa Ribeiro et al., 2013; Oltmanns et al., 2017; Curreri et al., 2022; Kleineidam et al., 2022). The same applies to chronic stress that causes anxiety and depression (Too et al., 2020), which in the longer term impairs almost all fluid functions (Lupien et al., 2007; Sterlemann et al., 2010; Marin et al., 2011).

Interventions within the framework of occupational health and competence management should therefore ideally address both the circumstances (e.g., work organization, reduction of stressors) and behavior [e.g., promotion of physical and mental activity or learning stress management competences (Flaxman and Bond, 2010; Lloyd et al., 2017)]. The results may be finally assessed by similar methods such as in the Occupational Health and Safety Management System (Karanikas et al., 2022).

Changes in cognitive functions with increasing age become particularly relevant in view of the challenges of the modern working world, which is characterized by the introduction of new technologies, high complexity of work, high work density, new spatial concepts with higher distraction (non-territorial offices), documentation obligations, and the increase of electronic communication and permanent accessibility (Bläsing et al., 2022). In addition to the potential challenges, cyber-physical systems, which play an important role in industry 4.0, offer various options for relieving the physical and mental strain on employees (Zolg et al., 2021). Digitization is also associated with a high need for qualifications. The necessary training usually does not take place in small companies, and when it does, older employees participate less frequently (Stara et al., 2020). It should be noted that increased environmental complexity is in principle beneficial for cognitive fitness, but can quickly overtax, especially in case of inadequately trained older employees, or when cognitive functions are impaired, for example, by many years of monotonous work (Andel et al., 2007, 2015; Marquie et al., 2010; Curreri et al., 2022). Also, a good fit between one’s own competences and work requirements has favorable effects on work performance and self-efficacy, while a discrepancy can generate stress (Ilmarinen, 2009, 2019; Trautmann et al., 2011).

Finally, the socioeconomic situation in factories can change within a very short period due to uncertain markets, supply chain disruptions, energy crisis, economic pressure, and strong international competition, as well as other unexpected events such as pandemics. This can lead to anxiety, existential insecurity, and fear of job loss, which can cause chronic stress among employees (Giorgi et al., 2020; Ganson et al., 2021; LaMontagne et al., 2021) that can be particularly detrimental as they age and may led to early retirement (van den Berg et al., 2010; Topa et al., 2017).

Overall, cognitive competences as well as abilities to cope with stress and overstraining situations are needed to handle work. The first important step in tackling this issue is to put occupational health and safety into practice and to provide preventive instruments for evaluating work-related stress in form of risk assessment. In addition, the program should consist of a company health management system that focuses on the promotion of cognitive and emotional competencies in addition to the aspects of occupational health.

### The precursor of the present study

The present study consisted of two parts, a diagnostic part, and an interventional part. Both were conducted in the same occupational context. The first part was designed as a cross-sectional study that examined the extent to which the type of work (repetitive vs. flexible) and age (young vs. older) were associated with changes in work-relevant fluid cognitive functions (Freude et al., 2010, 2012, 2013; Gajewski et al., 2010). About 90 younger and older blue-collar workers of a big car factory participated in the study to evaluate performance in cognitive tasks. Middle-aged and older employees with many years of repetitive work showed increased error rates in tasks with a high working memory load, while older employees with flexible work showed significantly lower error rates. In contrast, in tasks with a low working memory load, there were no performance differences between the flexibly and non-flexibly working older participants, suggesting no differences in basis psychomotor functions. These differences were supported by differences in measures of brain activity using EEG.

The findings confirm the results that long-term repetitive, mentally unchallenging work is negatively associated with performance in fluid cognitive functions, while flexible work ensures that these functions are largely intact even in older age (Schooler et al., 1999; Bosma et al., 2003; Andel et al., 2007, 2015; Marquie et al., 2010; Correa Ribeiro et al., 2013; Oltmanns et al., 2017; Curreri et al., 2022; Kleineidam et al., 2022). This could be due to an increase of mental capacities over time, establishing the so-called “cognitive reserve” which presumably decelerates age-related cognitive decline (Stern, 2009; Correa Ribeiro et al., 2013; Gajewski et al., 2020; Kleineidam et al., 2022). Moreover, the findings demonstrate which fluid functions are mainly affected by repetitive work, namely executive processes such as working memory, error detection and correction, which reflect the basics for learning.

In sum, the results indicate the need for cognitively demanding and varied work individually adapted to the capacities of the workers. Since this is not always achievable in practice, in the present study a short-term intervention was conducted to enhance cognitive and emotional skills among middle-aged and older employees with repetitive work.

### Purpose of the present study

Aim of the present study was to evaluate possible changes in fluid cognitive functions and stress resilience through multidimensional interventions in workers with repetitive work at assembly line in the same car factory as in the previous study (Freude et al., 2010, 2013; Gajewski et al., 2010). To our knowledge this is the first study addressing a trainer-guided cognitive and stress management trainings in an industrial setting. The study was designed as a randomized controlled intervention trial (RCT) with a wait list control group, and with pre-, post and follow-up measures. The follow-up testing was conducted to evaluate potential long-term effects of the cognitive training and to examine effects of combined stress management and cognitive training in the wait list control group applied after the passive phase. Beside general training effects on cognitive functions, we are interested in differential effects of cognitive training as a function of age (younger vs. older), shiftwork (shift work vs. nightshift) and baseline cognitive performance (low vs. high).

The study was conducted successively in two independent samples with a delay of one year. There were organizational reasons for this, for example, a limited number of participants who could be trained at the same time. Originally, we aimed pooling the samples together. However, there was a change in the general socioeconomic situation of the factory within the year in which the study was conducted, which had consequences for the employees and their employment prospects. Due to the company’s precarious situation, short-time work was introduced, but to different amount in Sample 1 and 2. During the training periods, the participants in Sample 1 were much more affected by short-time work and the associated negative consequences than those in Sample 2, who benefited from an adoption of a job security plan for a part of the workforce. Due to this unexpected situation, we had the opportunity to investigate the effectiveness of the training intervention also in relation to the workplace situation. Accordingly, we assume that stress level is higher, and the impact of stress-reducing training should be larger in Sample 1 than in Sample 2 due to higher occupational and existential fears. Moreover, the effects of the cognitive training should be less pronounced in Sample 1 due to lower motivation to participate in additional activities aside from the work.

In addition to stress-related questionnaires, objective measures of stress level and potential changes after interventions were assessed using cortisol awaking response (CAR; Kunz-Ebrecht et al., 2004; Stalder et al., 2016; Nicolson et al., 2020; Xiong et al., 2021). CAR is assumed to reflect a transducer of psychosocial and emotional experience into physiological activation and influences feelings of energy and physical well-being that may corroborate results of the subjective measures (Adam et al., 2006). In particular, the question was whether stress reduction occurs as result of different types of stress management trainings (e.g., Flaxman and Bond, 2010; Lloyd et al., 2017), and whether, in addition to the stress-related questionnaires, the subjective changes appear in changes of CAR. Moreover, the baseline difference in stress levels between Sample 1 and 2 due to the workplace situation should also be reflected in different levels of CAR. Due to the relaxation of the company’s socioeconomic situation and improvement of job perspectives of the employees in Sample 2, it was assumed that the participants of the latter sample would show reduced stress levels, higher motivation, and consequently enhanced mental capacities to achieve larger cognitive training effects. Thus, it was hypothesized that the impact of stress-reducing training should be larger, and the effects of cognitive training should be less pronounced in Sample 1 than in Sample 2. Thus, we propose that cognitive learning in middle-aged and older employees depends on the general stress level. In addition, it is important to clarify which specific cognitive functions are particularly vulnerable to stress.

## Materials and methods

### Participants

A sample of 120 blue-collar employees with repetitive work participated in the study. The participants were middle-aged and older employees who had carried out repetitive work for many years (mean 21 years). They were recruited from the same car factory where the initial, cross-sectional study had been conducted (Freude et al., 2010, 2013; Gajewski et al., 2010). The requirements for participation in the study were: age (40 years and older), no neurological or psychiatric diseases, and repetitive tasks in the production or assembly area that were confirmed by work council partners. After withdrawal of 4 participants due to personal or health reasons, the final sample consisting of 116 individuals (3 females) has been split randomly in two subsamples (due to organizational and logistic reasons). One half of the sample (Sample 1; n = 58, Age: 40–55 years, duration of the employment in the factory: *M* = 22.8, *SD* = 5.5) conducted the study first; the second half (Sample 2; n = 58, Age: 40–57 years, duration of the employment in the factory: *M* = 20.9, *SD* = 7.1) started the study after Sample 1 had finished the training and completed all measures (see Table 1 for demographics). The study was finished before the outbreak of the COVID-19 pandemic and was therefore not affected by any factor related to COVID-19. The study was approved by the local ethics committee of the Leibniz association in accordance with the declaration of Helsinki. All participants were informed about the purpose of the study and gave written informed consent.

**Table 1:** Demographic data of the Sample 1 and Sample 2.

Variable	COG	CTRL
*Sample 1*		
N	29	29
Age M (SD)	47.4 (3.95)	47.4 (3.69)
Gender (m/w)	27/2	28/1
Permanent night shift	9	7
Early and late shift	20	22
Education		
Primary	18	15
Secondary	8	9
high school diploma	2	3
Other	1	2

**Sample 2**		
N	26	32
Age M (SD)	47.0 (5.04)	46.1 (4.11)
Gender (m/w)	26/0	32/0
Permanent night shift	10	10
Early and late shift	16	22
Education		
Primary	19	15
Secondary	0	6
high school diploma	5	7
Other	2	4

COG, cognitive training group; CTRL, wait list control group.

### Characteristics of the factory and work tasks

Around 4,900 people were employed at the time the study was conducted. The number of employees from partner companies amounted to approximately 1,200. The factory consisted of 3 plants, one for production of two car models, a second for production of car axles and transmissions, and a third for worldwide shipment of spare parts. Various work shift models were implemented in the factory. The shift times were divided into the early shift (6:00 a.m. to 2:00 p.m.), the late shift (2:00 p.m. to 10:00 p.m.) and the night shift (10:00 p.m. to 6:00 a.m.). The night shift employees worked exclusively at night, while the other employees alternated between early and late shifts. Persons with cycle-related activities mainly carried out activities on the assembly line, which were characterized by a high proportion of monotonous and repetitive work under time pressure. A work cycle – the performance of one and the same activity – took 63 s on average. In this activity, the proportion of physical work was relatively high. The insertion of sheet metal into equipment, the installation of vehicle parts (e.g., seat belts, lights etc.), laying cables or screwing were specific tasks in this area. Overhead work and stooped postures were associated with these activities. Furthermore, painting work in assembly line production as well as simple quality checking were also typical activities.

### Study design

**Figure 1:**
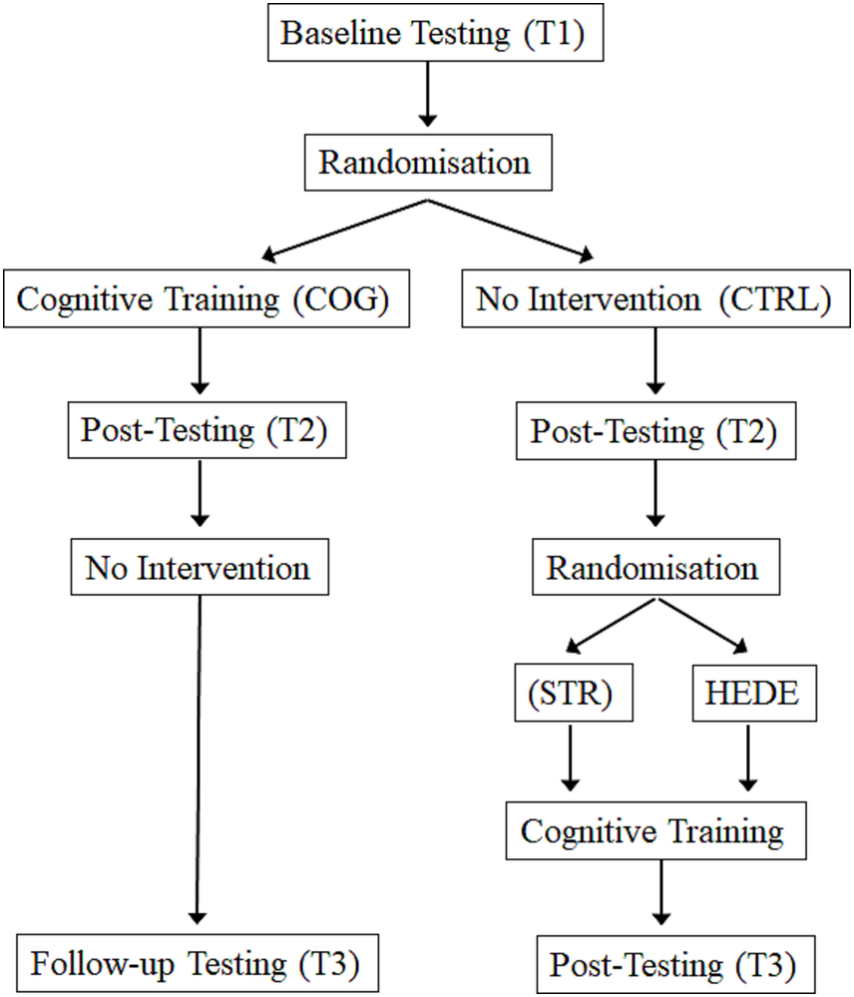
Design of the study. After the baseline testing (T1) the sample was randomly assigned to either pure cognitive training (COG) or the wait list control group (CTRL). After 3 months both groups completed a post-testing (T2). Whereas the COG group did not receive any intervention, the wait list control group was randomly assigned to either stress management group (STR) or psychological health program (HEDE) and absolved 8 sessions each before the cognitive training started for the remaining 12 sessions. After completing the training all participants were tested again. The design was the same for the Study Samples 1 and 2.

Figure 1 shows the design of the study with the chronological sequence of tests and trainings.

Each sample of participants was randomly divided into two subgroups at the beginning of the study: One half belonged to the training group, which participated in a purely cognitive intervention (COG) over a period of three months (20 sessions, 90 min per session, in total 30 h), the other half to the wait list control group (no-contact group; CTRL), which initially received no training. In the second step, after the training in the COG was finished, the participants of the wait list control group were randomly divided into two equal subgroups, each receiving qualitatively different stress-reducing interventions. The initial 8 sessions were conducted either with stress management training with relaxation (STR) or health promotion training (HEDE) before the cognitive training started. Thereafter participants received the same multidimensional cognitive training as the COG group that was shortened to 12 sessions so that again 20 sessions took place. Each training session was held either in the morning or in the afternoon to consider the different working hours of the participants.

Prior the initial randomization, the status of cognitive functions was examined (baseline, Session T1). The same examination took place after completion of the cognitive training, i.e., after approx. 3 months (T2) and after completion of the combined training, i.e., after approx. 6 months (T3). This follow-up measure made it possible to observe the sustainability of cognitive changes after the end of the training in the group with the pure cognitive training, and to evaluate the effects of the combined training. The same training interventions and experimental design were used in both samples.

### Training contents

The training was offered as a health-promoting activity and took place in the department of occupational health and safety in the factory. All trainings were carried out by professional trainers certified by the German Brain Training Association (Gesellschaft für Gehirntraining e.V. (GfG)) who were paid for their service. To enhance adherence to the training it was conducted in the training rooms of the company in the department of safety and occupational health.

As mentioned above one group was given purely cognitive training, whereas the wait list control group later received a combination of a cognitive training (with reduced number of sessions) and a stress management or psychological health promotion training.

### Cognitive training

Within the cognitive training fluid cognitive functions were trained like working and verbal memory, executive functions, processing speed, selective, sustained attention, spatial attention, and logical thinking. The training program consisted of various paper- and PC-based tasks to activate these functions in as many contexts as possible (multidimensional training), Gajewski et al. (2020). This procedure should promote the far-transfer, i.e., the improvement of competences also in other tasks and work activities that require the trained functions. The trainings were selected from various paper and pencil templates and commercial PC programs according to the criteria meaningfulness, i.e., promoting well-known cognitive functions like different types of memory or attention, and fun factor to enhance the motivation and persistence to train.

The schedule of each session was as follow: 1. Two or three PC-based exercises, 2. theoretical part, 3. short relaxation (3–5 min), 4. paper-pencil exercises, 5. two or three PC-based exercises (please refer to Supplementary material for description of the training contents in more detail).

The participants were able to determine the level of difficulty according to their daily form and motivation, which turned out to be an important factor for a successful participation. At the same time, the participants received information about changes in cognitive abilities with increasing age and potentials of cognitive training to reduce cognitive decline to increase their motivation to maintain training even after the end of the study.

The cognitive training groups were divided into two sub-groups, which differed in terms of learning speed. In this way it was possible to prevent the participants from being over- or underchallenged. This had a particularly positive effect on the group of weaker participants: The more intensive contact with the trainer led to greater acceptance of the training. This enhanced the motivation of participants.

### Stress management trainings

Prior the cognitive training sessions the employees of the wait list control group participated in eight sessions of stress management training. There were two different types of training practiced in two subgroups. One was a variant of the stress management training (STR) according to Beck (1980), Jacobson (2017), and Kaluza (2018), consisting of theoretical information, practical exercises, and interactive group work.

In the STR group the role of dysfunctional thoughts in stressful situations was highlighted. Subsequently, these dysfunctional thoughts were transformed into stress-reducing actions. This theoretical part was followed by muscle relaxation training (Progressive Muscle Relaxation (PMR); 61). The technique involves learning to monitor the tension and relaxation in specific muscle groups.

The other variant was a training for psychological health promotion program (HEDE training^®^; 63), which is based on the concept of salutogenesis according to Antonovsky (1987) and Mittelmark et al. (2018). The salutogenetic model examines the question why people–despite stress and health-threatening stimuli – remain healthy. The theory proposes existence of a “generalized resistance resources” as the factors that contribute decisively to a constructive handling of stressors. As a superordinate personal resource, the training aims at strengthening the “sense of coherence,” which encompasses the elements of comprehensibility, manageability, and significance through the development of resource-promoting coping behavior.

Please refer to Supplementary material for detailed description of the stress management trainings.

### Testing

After the telephone interview and successful appointment, participants received a series of questionnaires asking for sociodemographic characteristics, work-related aspects, stress experience, and health-related experience. Training-related questionnaires were used after the training to evaluate the training programs as feedback for the trainer. The participants filled out all questionnaires out at home and gave them back at the testing day.

### Psychometric testing

Psychometric tests were used to evaluate cognitive changes in the trained areas. The tests were performed for the training and wait list control groups in a double blinded design, as the training and control group randomization took place after the baseline measure was finished. All subjects were tested within four weeks before and after the training. The tests were performed at the Leibniz Institute for Working Environment and Human Factors (IfADo) at the same time of day and by the same examiners and lasted approximately 90 min each.

The assessment was conducted with psychometric paper and pencil tests using well-established, standardized test batteries measuring a broad spectrum of cognitive abilities such as sustained and focused attention (d2; Brickenkamp, 1998), crystalized intelligence (LPS-3), verbal fluency (LPS-6) and mental rotation (LPS-7) using subtests from the performance test system (LPS; Horn, 1983). Short- and working memory were assessed using digit-span forward (DS-F) and backward (DS-B) tests, interference processing with the Stroop test, and psychomotor speed was measured with digit-symbol-test (DST) from the Nürnberg Age Inventory (NAI; Oswald and Fleischmann, 1999). Different aspects of verbal short and long-term memory were measured with the Verbal Learning and Memory Test (VLMT; Helmstaedter et al., 2001). Divided attention between visual and auditive modalities was measured by the attention test battery (TAP; Zimmermann and Fimm, 2002) and psychomotor speed and task switching were evaluated by using the Trail-Making Test (TMT; Reitan, 1992). In the post-measurement, most tests used parallel versions with the same structure and level of difficulty, but different content. Please refer to Supplementary material for detailed description of the psychometric tests.

After the psychometric testing session, several PC-based executive tasks with EEG recording were applied using the same tasks as in the precursor, diagnostic study (Gajewski et al., 2010). The data were published elsewhere (Gajewski et al., 2017). The participants were paid for participation in multiple cognitive testing at IfADo.

### Questionnaires

Several questionnaires were included to assess sociodemographic, lifestyle, and individual factors as well as changes in the cognitive, emotional and stress-related aspects due to training. Self-reported mental and physical capacities at work were assessed by the work ability index at baseline in all participants (WAI), (Ilmarinen, 2006). Sense of Coherence Questionnaire (SOC), (Antonovsky, 1987) was used to evaluate the effectiveness of the HEDE-training^®^ (HEDE), (Franke and Witte, 2009). General Health Questionnaire (GHQ-12), (Goldberg and Williams, 1991) measures the risk for the development of a psychiatric disorder and psychological distress in the past two weeks. Questions on physical well-being were used to assess current physical well-being and positive a positive body feeling (Franke and Witte, 2009). The questionnaires assessing the effectiveness of the HEDE-Training were only filled out by the participants of the HEDE-Group.

The questionnaire on work-related behavior and experience patterns (AVEM) (Schaarschmidt and Fischer, 2008) was used to test the training effects in the occupational context. This is a standardized diagnostic procedure for recording behavior and experience in relation to work and occupational requirements from the health-relevant perspective. The Perceived Stress Questionnaire (PSQ-20), (Levenstein et al., 1993) consists of four dimensions (worry, tension, pleasure and demands) and was used to assess the subjective perception, evaluation, and processing of stressors. MBI (Maslach and Jackson, 1981; Maslach, 1997) is a valid instrument for multidimensional assessment of burnout. It represents the only instrument that captures all core dimensions of burnout: emotional exhaustion, reduced efficiency, and depersonalization. The Cognitive Failures Questionnaire (CFQ), (Broadbent et al., 1982) is a questionnaire used for self-assessment of failures in perception, memory, and motor functions in daily life. Broadbent et al. (1982) suggest that the instrument may capture a general deficit of cognitive control and that a high CFQ score is associated with reduced stress resistance. Please refer to Supplementary material for detailed description of the questionnaires.

Several questions were asked about the participants’ satisfaction with the training. On the one hand, they were asked whether participation was considered a success (four-stage answer format “success”–“failure”). Moreover, they were asked more globally how satisfied they were with the training (three-stage response format “very satisfied”–“dissatisfied”). An assessment was based on the school grading system (1: very good, 6: insufficient). A yes/no question was asked as to whether the participants would recommend the training to others.

### Cortisol awakening response

The CAR is an index of the level of psychosocial, emotional, and physical stress and is used to translate psychological and emotional experiences into physiological activation (Adam et al., 2006).

To measure CAR, a saliva sample was collected immediately upon awakening and a second sample was collected 30 min later when the concentration is highest. The changes associated with the current mood state are most evident in the second saliva sample and the data quality depends on the compliance and reliability of the subjects and the extent to which they adhered to the conditions of the survey (Kunz-Ebrecht et al., 2004; Stalder et al., 2016). It was not allowed to eat, drink or smoke between awaking and the second sample.

### Data analysis

All personal data were anonymized, and all analyses were conducted using a code. Descriptive statistics, frequencies, and distributions were calculated for all quantitative variables.

In the pure cognitive training group (COG) and the control group (CTRL) effects of cognitive training were evaluated with two-way analyses of variance with repeated measures (mixed ANOVA). The factor Session [pre-measurement (T1) vs. post-measurement (T2)] was the within-subject factor, and the Group (training group (COG) vs. wait list control group (CTRL) was the between-subject factor. If the interaction measurement Session × Group became significant, subsequent analyses of variance were performed separately for both groups to provide information about the origin of the interaction. If significant group differences in the cognitive variables were present at T1, the differences between the training and wait list control groups from the post- and pre-measurement were compared. Later when the control group was also trained effects of the stress reduction training (STR) and the psychological health program (HEDE) were tested between T2 and T3. Here, the ANOVA included the within-subject factor Session (T2 vs. T3) and between-subject factor Group (STR vs. HEDE). The analysis was also applied for the cortisol awakening response (CAR), but additionally included the factor Saliva Sample (immediately after awaking vs. 30 min later).

A prerequisite for the unambiguous interpretation of effects of cognitive intervention measures is to ensure groups with the same baseline conditions in demographic and cognitive variables, and in cognitive performance levels. In addition to examining group differences in the variables age and education (*cf.* Table 1), the comparability of the two groups (training group/wait list control group) regarding cognitive baseline profile was tested. For this purpose, T-tests for independent samples or–if the presumptions for a *T*-test were violated–Mann–Whitney-U or a non-parametric Wilcoxon test for connected samples were used. Some rare missing values in the questionnaire data were calculated by multiple imputation. Data were analyzed using the Statistical Package for Social Sciences (SPSS; IBM Statistics, Version 28).

Data of Samples 1 and 2 were analyzed and reported separately.

## Results

### Work Ability Index

In Sample 1 the mean scores in the WAI at baseline (T1) were *M* = 35.66, *SD* = 6.04 in the COG group, *M* = 34.64, *SD* = 4.34 in the CTRL, later STR subgroup, and *M* = 33.53, *SD* = 6.54 in the CTRL later HEDE subgroup with no group differences (*F* < 1). The average score was *M =* 34.86, *SD* = 5.77, suggesting a moderate work ability.

Sample 2 showed slightly higher scores: *M* = 38.54, *SD* = 6.12 in the COG group, *M* = 36.00, *SD* = 7.58 in the CTRL, later STR subgroup, and *M* = 33.63, *SD* = 8.25 in the CTRL, later HEDE subgroup with no group differences (*F* < 1), and an average score of *M* = 36.46, being at the border between moderate and good work ability.

### Effects of cognitive interventions

In the following different aspects of the cognitive intervention are presented. First, the cognitive performance between the samples at baseline, effects of pure cognitive training in Sample 1 and 2 (T1 vs. T2), the analysis of the follow-up measures (T2 vs. T3), differential effects of training as a function of age, baseline performance and shift (T1 vs. T2), comparison between the purely cognitive training (T1 vs. T2) and the combined intervention (T2 vs. T3).

With one exception, all participants rated their participation as a success. The same was true for the satisfaction with the entire training, with 99% of the participants recommending the training to others.

Please note that statistical results included in the tables were not included in the text. Additional results not included in the tables were described in the text including descriptive and inference statistics.

### Cognitive performance at baseline

To interpret cognitive changes between T1 and T2 as an effect of the intervention, the dependent variables in the COG and CTRL groups should not differ at T1.

In respect to the cognitive variables, the comparison of baseline measures in Sample 1 between the COG and the CTRL groups revealed that CTRL showed a superior performance in only one parameter (see Supplementary Tables 1, 2 in Supplementary material); the total number of correctly retrieved words in trials 1 to 5 in the VLMT test. Here, CTRL had a higher mean than COG. For Sample 2, only a difference in the sum of errors in the attentional endurance test (d2) was found. The training group showed a higher mean value than the wait list control group. No further baseline differences were found.

### Effects of pure cognitive training (T1 vs. T2)

Sample 1 showed only rare effects of cognitive training (Table 2). In the Digit-Symbol- Test (DST) the ANOVA revealed an interaction Session × Group, which was based on an improvement of the participants in the training group, but not the control group. Additionally, recognition performance in the VLMT test yielded an interaction Session × Group, which was based on an trend for improvement of the participants in the COG group and no changes in the CTRL group. No further interactions were found. Supplementary Table 3 in Supplementary material presents descriptive statistics in of the tests at T1 and T2.

**Table 2:** Training effects in cognitive tests in Sample 1.

Variable	Group	Difference T2-T1	ANOVA between groups				ANOVA within groups			
		ΔM (SD)	*F*	*df*	*p*	*d*	*F*	*df*	*p*	*d*
*Attentional endurance test (d2)*										
Total number of symbols	COG CTRL	20.17 (55.37) 9.76 (55.24)	< 1	1.56	n.s.		3.849 < 1	1.28 1.28	0.06 n.s.	−0.26
Total Number of correct symbols	COG CTRL	23.76 (54.85) 19.52(49.07)	< 1	1.56	n.s.		5.442 4.588	1.28 1.28	0.027 0.041	−0.34 −0.32
Total number of errors^1.2^	COG CTRL	−3.59 (10.79) −9.45 (17.39)	2.380	1.56	0.129	0.24	3.202 8.565	1.28 1.28	0.084 0.007	0.13 0.45
Concentration performance	COG CTRL	9.52 (16.71) 12.28 (14.41)	< 1	1.56	n.s.		9.406 21.055	1.28 1.28	0.005 <0.001	−0.30 −0.52
*Performance Testing System (LPS)*										
LPS-1	COG CTRL	0.07 (4.31) −0.34 (2.61)	< 1	1.56	n.s.		< 1 < 1	1.28 1.28	n.s. n.s.	
LPS-3	COG CTRL	2.62 (3.21) 2.55 (3.51)	< 1	1.56	n.s.		19.309 15.317	1.28 1.28	<0.001 0.001	−0.70 −0.56
LPS-6	COG CTRL	0.89 (6.39) 2.21 (4.30)	< 1	1.56	n.s.		< 1 7.624	1.28 1.28	n.s. 0.01	−0.27
LPS-7	COG CTRL	4.14 (3.45) 2.59 (5.22)	1.783	1.56	0.187	0.28	41.696 7.118	1.28 1.28	<0.001 0.013	−0.78 −0.47
*Nürnberg Age Inventory (NAI)*										
digit-span forward	COG CTRL	−0.10 (1.05) −0.10 (1.11)	< 1	1.56	n.s.		< 1 < 1	1.28 1.28	n.s. n.s.	
digit-span backward	COG CTRL	0.07 (1.33) 0.21 (1.49)	< 1	1.56	n.s.		< 1 < 1	1.28 1.28	n.s. n.s.	
Digit-Symbol-Test	COG CTRL	4.10 (5.28) 1.76 (7.52)	**6.290**	**1.43**	**0.016**	0.29	17.514 1.584	1.28 1.28	<0.001 0.219	−0.66 −0.20
Stroop (s)^2^	COG CTRL	−1.83 (5.64) −1.85 (5.57)	< 1	1.54	n.s.		3.047 2.982	1.28 1.26	0.092 0.096	0.26 0.22
Stroop difference (s)^2^	COG CTRL	−0.97 (5.74) −1.44 (5.39)	< 1	1.56	n.s.		< 1 1.941	1.28 1.26	n.s. 0.175	0.24
*Test Battery for Attention Assessment (TAP)*										
Auditive condition (ms)^2^	COG CTRL	−9.22 (78.50) −5.89 (55.69)	< 1	1.54	n.s.		< 1 < 1	1.26 1.28	n.s. n.s.	
Visual condition (ms)^2^	COG CTRL	−13.36 (123.51) −45.62 (79.09)	1.389	1.55	0.244	0.26	< 1 9.649	1.27 1.28	n.s. 0.004	0.37
Total number of errors^1.2^	COG CTRL	−1.19 (3.25) −0.03 (2.02)	2.570	1.53	0.115	−0.39	3.500 < 1	1.25 1.28	0.073 n.s.	0.34
*Verbal Learning and Memory Test (VLMT)*										
Total number of words	COG CTRL	4.34 (6.59) 4.00 (7.06)	< 1	1.56	n.s.		12.600 9.320	1.28 1.28	0.001 0.005	−0.50 −0.54
Immediate reproduction^2^	COG CTRL	−0.55 (2.15) −0.17 (1.79)	< 1	1.56	n.s.		1.914 < 1	1.28 1.28	0.178 n.s.	0.31
Delayed reproduction^2^	COG CTRL	−0.55 (2.31) 0.28 (2.33)	1.848	1.56	0.179	−0.44	1.657 < 1	1.28 1.28	0.209 n.s.	0.25
Recognition performance	COG CTRL	1.14 (3.04) −0.52 (2.61)	**4.900**	**1.55**	**0.031**	0.55	3.959 1.136	1.27 1.28	0.057 0.296	−0.34 0.20
*Trail Making Test (TMT)*										
Version A	COG CTRL	−4.79 (9.29) −3.97 (5.34)	< 1	1.56	n.s.		7.713 16.022	1.28 1.28	0.01 <0.001	0.48 0.46
Version B	COG CTRL	−8.31 (17.05) −4.72 (19.54)	< 1	1.56	n.s.		6.887 1.695	1.28 1.28	0.014 0.204	0.43 0.20
Difference B–A	COG CTRL	−3.52 (18.58) −0.76 (19.58)	< 1	1.56	n.s.		1.039 < 1	1.28 1.28	0.317 n.s.	0.19
*Cognitive Failures Questionnaire (CFQ)*										
Total score	COG CTRL	−2.41 (8.03) −0.52 (6.29)	1.001	1.56	0.321	−0.17	2.616 < 1	1.28 1.28	0.117 n.s.	0.27

Tests for differences (T2 – T1) in psychometric tests for the cognitive training group (COG; *N* = 29) and the wait list control group (CTRL; *N* = 29). The mean differences (ΔM) were compared between the groups, and the values at T1 and T2 were compared within groups. *N*, sample size; *M*, mean; *SD*, standard deviation; *F*, *F*-value; *df*, degree of freedom; *p*, significance level; *d*, effect size. Significant values are printed in bold.

Sample 2 showed more consistent effects of cognitive training than Sample 1 (Table 3).

**Table 3:** Training effects in cognitive tests in Sample 2.

Variable	Group	Difference T2-T1	ANOVA between groups				ANOVA within groups			
		ΔM (SD)	*F*	*df*	*p*	*d*	*F*	*df*	*p*	*d*
*Attentional endurance test (d2)*										
Total number of symbols	COG CTRL	19.04 (50.09) 22.22 (46.64)	< 1	1.56	n.s.		3.757 7.262	1.25 1.31	0.064 0.011	−0.27 −0.33
Total Number of correct symbols	COG CTRL	21.69 (36.05) 22.84 (44.82)	< 1	1.56	n.s.		9.412 8.312	1.25 1.31	0.005 0.007	−0.32 −0.33
Total number of errors	COG CTRL	−2.65 (30.31) −0.03 (17.48)	< 1	1.56	n.s.		< 1 < 1	1.25 1.31	n.s. n.s.	
Concentration performance	COG CTRL	11.62 (22.64) 10.88 (23.56)	< 1	1.56	n.s.		6.842 6.816	1.25 1.31	0.015 0.014	−0.35 −0.31
*Performance Testing System (LPS)*										
LPS-1	COG CTRL	0.00 (3.86) −0.75 (3.82)	< 1	1.56	n.s.		< 1 1.235	1.25 1.31	n.s. 0.275	0.13
LPS-3	COG CTRL	3.19 (3.60) 2.06 (3.45)	1.482	1.56	0.229	0.24	20.442 11.471	1.25 1.31	<0.001 0.002	−0.73 −0.44
LPS-6	COG CTRL	4.00 (4.89) 1.19 (4.72)	**4.922**	**1.56**	**0.031**	0.38	17.333 2.025	1.25 1.31	<0.001 0.165	−0.56 −0.16
LPS-7	COG CTRL	5.77 (6.38) 3.53 (4.34)	2.514	1.56	0.118	0.35	21.281 21.183	1.25 1.31	<0.001 <0.001	−0.91 −0.55
*Nürnberg Age Inventory (NAI)*										
digit-span forward	COG CTRL	0.54 (1.30) −0.28 (1.28)	**5.808**	**1.56**	**0.019**	0.80	4.438 1.555	1.25 1.31	0.045 0.222	−0.59 0.26
digit-span backward	COG CTRL	0.15 (1.29) 0.34 (1.41)	< 1	1.56	n.s		< 1 1.915	1.25 1.31	n.s. 0.176	−0.34
Digit-Symbol-Test	COG CTRL	−0.15 (6.10) −3.00 (7.11)	2.606	1.56	0.112	0.28	< 1 5.701	1.25 1.31	n.s. 0.023	0.26
Stroop (s)	COG CTRL	−3.42 (6.59) −0.94 (5.39)	2.458	1.55	0.123	−0.29	6.998 < 1	1.25 1.30	0.014 n.s.	0.38
Stroop difference (s)	COG CTRL	−2.77 (6.72) −0.94 (5.16)	1.357	1.55	0.249	−0.28	4.417 1.020	1.25 1.30	0.046 0.321	0.38 0.16
*Test Battery for Attention Assessment (TAP)*										
Auditive condition (ms)^2^	COG CTRL	−17.19 (83.96) 12.19 (69.93)	2.115	1.56	0.151	−0.34	1.090 < 1	1.25 1.31	0.306 n.s.	0.18
Visual condition (ms)^2^	COG CTRL	−40.20 (96.54) −24.22 (108.57)	< 1	1.55	n.s.		4.335 1.592	1.24 1.31	0.048 0.216	0.55 0.25
Total number of errors	COG CTRL	−2.69 (5.02) −0.63 (4.84)	**5.101**	**1.42**	**0.029**	−0.44	7.484 < 1	1.25 1.31	0.011 n.s.	0.55
*Verbal Learning and Memory Test (VLMT)*										
Total number of words	COG CTRL	3.19 (7.04) 2.41 (7.80)	< 1	1.56	n.s.		5.350 3.043	1.25 1.31	0.029 0.091	−0.51 −0.36
Immediate reproduction	COG CTRL	0.15 (2.05) 0.53 (2.62)	< 1	1.56	n.s.		< 1 1.321	1.25 1.31	n.s. 0.259	
Delayed reproduction	COG CTRL	−0.08 (1.47) 0.41 (2.03)	1.033	1.56	0.314	−0.32	< 1 1.282	1.25 1.31	n.s. 0.266	−0.25
Recognition performance	COG CTRL	0.19 (1.58) −0.28 (3.08)	< 1	1.56	n.s.		< 1 < 1	1.25 1.31	n.s. n.s.	
*Trail Making Test (TMT)*										
Version A	COG CTRL	−4.54 (7.52) −2.69 (8.39)	< 1	1.56	n.s.		9.479 3.276	1.25 1.31	0.005 0.08	0.62 0.26
Version B	COG CTRL	−17.69 (24.41) −6.03 (15.99)	**4.786**	**1.56**	**0.033**	−0.43	13.656 4.553	1.25 1.31	0.001 0.041	0.58 0.26
Difference B–A	COG CTRL	−13.15 (24.26) −3.34 (13.47)	**3.801**	**1.56**	**0.056**	−0.42	7.645 1.971	1.25 1.31	0.011 0.170	0.47 0.18
*Cognitive Failures Questionnaire (CFQ)*										
Total score	COG CTRL	−0.18 (8.13) 0.47 (7.03)	< 1	1.56	n.s.		< 1 < 1	1.25 1.31	n.s. n.s.	

Tests for differences (T2 – T1) in psychometric tests for the cognitive training group (COG; *N* = 26) and the wait list control group (CTRL; *N* = 32). The mean differences (ΔM) were compared between the groups, and the values at T1 and T2 were compared within groups. *N* = sample size; *M* = mean; *SD* = standard deviation; *F* = *F*-value; *df* = degree of freedom; *p* = significance level; *d* = effect size. Significant values are printed in bold.

As evident from Table 3 the analysis of word fluency (LPS 6) revealed an interaction Session × Group, due to an improvement in the training group. The COG was able to generate an average four more words after the intervention, while the CTRL improved by an average of one word. For TMT-B, there was a significant interaction Session × Group: The COG was faster by an average of 17 s after the intervention. The CTRL showed an improvement in performance of 6 s. For the difference TMT B-A, there was a trend for the interaction, which was due to a performance enhancement in the COG group that was faster after the intervention by 14 s, while the CTRL group was faster by 3 s. For the digit-span forward, the analysis yielded an interaction Session × Group, which was due to a small but substantial improvement in the COG group, while performance remained unchanged in the CTRL group. Also, the number of errors on the auditory and visual task (TAP) showed an interaction that was due to less errors in the COG group after training, whereas there was no significant change in the CTRL group. Supplementary Table 4 in Supplementary material presents descriptive statistics of the tests at T1 and T2 in Sample 2.

In summary, in Sample 1 the evaluation of the cognitive intervention revealed substantial training gains in one test measuring psychomotor speed and focused attention (DST; digit-symbol test) and showed a trend for improved recognition performance (VLMT). Sample 2 showed more consistent gains: in the word fluency and verbal flexibility (LPS 6), in the attentional processes measured by TAP, for short-term memory (DS-F; digit-span forward), psychomotor speed and focused attention (DST; digit-symbol test), and for task switching as an executive process (TMT-B and a trend for the difference TMT-B–TMT-A).

### Analysis of the follow-up measure in the COG Group (T2 vs. T3)

The COG group of Sample 1 showed an increase in performance between T2 and T3 for the concentration performance (*F*(1, 22) = 8.48; *p* = 0.008), the total number of correct symbols (*F*(1, 21) = 4.87; *p* = 0.038), and a lower number of errors (*F*(1, 21) = 4.99; *p* = 0.036). The digit-symbol-test showed an increase of correctly performed symbols from T2 to T3 (*F*(1, 21) = 4.43; *p* = 0.048), while the number of errors in the auditory and visual task (TAP) was reduced (*F*(1, 19) = 4.64; *p* = 0.044). For the interference condition of the Stroop test (Stroop 3), there was a trend for faster task completion at T3 compared to T2 (*F*(1, 22) = 3.80; *p* = 0.064). For LPS-3, participants performed more symbols at T3 than at T2 (*F*(1, 22) = 5.92; *p* = 0.024). For the corrected recognition performance (VLMT), the mean score decreased at T3 (*F*(1, 20) = 5.70; *p* = 0.027).

In contrast, in Sample 2 no further changes occurred in between T2 vs. T3. Only the LPS-7 showed an effect (*F*(1, 19) = 5.72; *p* = 0.027) due to performance decrease.

In summary, whereas Sample 1 showed hardly any training effects directly after the intervention, performance was improved three months after the end of the intervention. In contrast, Sample 2 demonstrating improvements immediately after the intervention showed no further improvement three months after the intervention.

### Differential training effects (T1 vs. T2)

To evaluate differential effects of cognitive training as a function of age, shift work, and cognitive baseline level, ANOVAs with the within-subject factor Session (T1 vs. T2) and the between-subject factors Group (COG vs. CTRL), Age (young/old), Shift Type (permanent night work/early and late shift), and Baseline Performance (low/high) were conducted. Both factors Age and Baseline Performance were included in the analysis using median split to dichotomize the variables. The median age in Sample 1 was 47 years (40–55 years old), and in Sample 2, 46 years (40–57 years old). Significant results were included in Supplementary material.

In summary, in both samples, the largest training-related gains were observed in night shift workers who showed a low performance at baseline in the executive attention (TAP) and logical reasoning (LPS-3). However, in general differential effects occurred only rarely. This finding is to be considered as positive, because cognitive training should provide benefit for all participants, regardless of age, type of shift or baseline level.

### Comparison between the purely cognitive training (T1 vs. T2) and the combined intervention (T2 vs. T3)

The comparison of efficacy between the purely cognitive intervention (COG) and the combined training after the passive control phase (STR/HEDE + COG) was conducted only descriptively. The participants of the wait list control group had already undergone two psychometric sessions (T1 and T2) at the beginning of their STR/HEDE + COG training, whereas the intervention in the COG group started after the baseline measurement (T1). Since performance in psychometric tests can improve due to repeated testing despite parallel versions, it must be assumed that there is a confounding of training and repetition effects.

For this purpose, averaged differences before and after the respective training (COG: T2 – T1; STR/HEDE + COG: T3 – T2) were compared descriptively (see Supplementary material).

In summary, while both COG and STR/HEDE + COG groups improved in an equal number of domains in Sample 1, the participants in the COG group in Sample 2 showed performance gains in more domains than the participants in the combined intervention. Importantly, there was a greater improvement in both Sample 1 and 2 in perception of everyday cognitive failures and inattentiveness (CFQ) in the STR/HEDE + COG than the COG groups. The assessment of cognitive inattentiveness can be taken as an indication of the changes in everyday life after the combined intervention and suggest a far-transfer. Whether this effect was due to the combination of stress management training and cognitive training or to the stress-related component alone cannot be conclusively answered. However, it has been suggested that CFQ scores did not predict performance on objective neuropsychological tests but were related to a range of psychological stress symptoms, as originally suggested by Broadbent et al. (1982). This might indicate that stress-management training reduces daily inattentiveness by an increased stress resilience rather than by cognitive training.

### Effects of stress management interventions (T2 vs. T3)

After the passive control phase between T1 and T2, participants of the wait list control group (CTRL) received a combination of stress management training and cognitive intervention between T2 and T3. Half of the participants participated in the stress management training (STR). The other half received the HEDE-Training^®^ (Franke and Witte, 2009). These participants are referred to as the HEDE group. The training arrangements were the same for Sample 1 and Sample 2. The effectiveness of the two stress-related interventions was assessed by using questionnaires before and after the interventions and compared between the two types of interventions and both samples. Furthermore, changes of the cortisol awakening response (CAR) between T2 and T3 are presented for Sample 1 and 2. Finally, baseline differences between the samples in the stress-related parameters and CAR are analyzed.

### HEDE-training^®^

Table 4 summarizes the mean scores of the HEDE Training effectiveness questionnaire before and after the intervention. A significant difference was found between T2 and T3 in four out of 11 variables: For the General Health Questionnaire (GHQ) the mean value before the start of training indicated increased distress, while after training it was reduced to a non-critical value (*F*(1, 14) = 4.58; *p* = 0.05). Also, the score of the physical well-being scale improved (*F*(1, 14) = 9.51; *p* = 0.008). For coping with partnership concerns, there was also a significant improvement. The mean score for task management at work increased after HEDE Training.

**Table 4:** Effects of the HEDE training on different variables of the HEDE questionnaire in Sample 1.

Variable/Dimension	*N*	T2 *M (SD)*	T3 *M (SD)*	*p*
Sense of Coherence Questionnaire (SOC-13)	15	4.63 (0.83)	4.72 (0.84)	n.s.
Score GHQ	15	13.07 (7.61)	10.11 (6.38)	**0.050**
Score physical well-being	15	16.07 (4.54)	20.64 (4.84)	**0.008**
Task management in the partnership^1^	12	1.83 (0.39)	2.42 (0.52)	**0.016**
Coping with tasks in dealing with children^1^	12	1.83 (0.39)	2.25 (0.45)	n.s.
Task management in contact with friends^1^	12	1.83 (0.58)	2.08 (0.29)	n.s.
Coping with tasks at work^1^	12	1.75 (0.45)	2.33 (0.49)	**0.031**
Coping with tasks in contact with authorities/offices^1^	12	1.83 (0.39)	2.08 (0.52)	n.s.
Coping with tasks in contact with strangers^1^	12	2.25 (0.45)	2.42 (0.52)	n.s.
Coping with tasks in hobbies^1^	12	2.08 (0.67)	2.25 (0.62)	n.s.
Coping with tasks in contact with therapists/doctors^1^	12	2.17 (0.58)	2.25 (0.45)	n.s.

*N* = sample size; *M* = mean; *SD* = standard deviation; *p* = significance level.

1 Wilcoxon-Test for connected samples. Significant values are printed in bold.

Table 5 shows the mean scores of the questionnaire evaluating the HEDE Training before and after the intervention in the Sample 2. There was a trend toward significant difference between pre- and post-measurement on the General Health Questionnaire (GHQ) (*F*(1, 14) = 4.57; *p* = 0.051). The mean score of at baseline increased after training, indicating an increase in psychological distress. In summary, only 1 out of 11 variables showed trend for improvement due to the HEDE-training in sample 2.

**Table 5:** Dimensions of the questionnaire for testing the effectiveness of the HEDE training in Sample 2.

Variable/Dimension	*N*	T2 *M (SD)*	T3 *M (SD)*	*p*
Sense of Coherence Questionnaire (SOC-13)	15	4.68 (0.65)	4.54 (0.53)	n.s.
Score GHQ	15	10.60 (6.02)	13.33 (8.33)	0.051
Score physical well-being	15	17.20 (5.76)	16.73 (3.67)	n.s.
Task management in the partnership^1^	12	1.75 (0.62)	1.83 (0.72)	n.s.
Coping with tasks in dealing with children^1^	14	1.93 (0.73)	2.21 (0.43)	n.s.
Task management in contact with friends^1^	14	1.93 (0.48)	1.64 (0.75)	n.s.
Coping with tasks at work^1^	15	1.80 (0.68)	1.93 (0.59)	n.s.
Coping with tasks in contact with authorities/offices^1^	9	1.78 (0.44)	1.78 (0.44)	n.s.
Coping with tasks in contact with strangers^1^	13	2.00 (0.58)	2.08 (0.28)	n.s.
Coping with tasks in hobbies^1^	13	1.69 (0.63)	1.77 (0.93)	n.s.
Coping with tasks in contact with therapists/doctors^1^	8	1.75 (0.46)	2.00 (0.54)	n.s.

*N*, sample size; *M*, mean; SD, standard deviation; *p*, significance level.

1 Wilcoxon-Test for connected samples.

### Comparison between stress management and psychological health promotion

For the comparison of the two stress-related interventions (STR and HEDE), ANOVAs with the factors Session (T2 vs. T3) and Group (STR/HEDE) were conducted. Furthermore, within the groups one-way ANOVAs for the factor Session were conducted. Differences between post and pre-measurement (T3 – T2) were compared between the two groups. Tables 6, 7 show the demographic data of the two subgroups and Sample 1 and Sample 2.

**Table 6:** Demographic data of participants in the stress-related interventions in Sample 1.

Variable	STR	HEDE	*p*
*N*	14	15	
Age *M* (SD)	46.64 (2.56)	48.07 (4.48)	n.s.
Sex (m/w)	13/1	15/0	
*Education*			
no	1	0	
Primary	5	9	
Secondary	7	2	
High school diploma	1	2	
other	0	2	

STR, Stress management training; HEDE, psychological health promotion training; *N*, sample size; *M*, mean; *SD*, standard deviation; *p*, significance level.

**Table 7:** Demographic data of participants in the stress-related interventions in Sample 2.

Variable	STR	HEDE	*p*
*N*	16	15	
Age M (SD)	46.94 (4.91)	45.27 (3.15)	n.s.
Sex (m/w)	16/0	15/0	
*Education*			
no	0	0	
Primary	9	5	
Secondary	4	2	
High school diploma	3	4	
other	2	2	

STR, Stress management training; HEDE, psychological health promotion training; *N*, sample size; *M*, mean; *SD*, standard deviation; *p*, significance level.

Table 8 provides an overview of the effects in stress measuring questionnaires in Sample 1. The analysis for the scale “inner calm and balance” (AVEM) revealed an interaction of Session × Group, which was due to an increase in experienced inner calm only in the HEDE group after training. Additionally, there was a main effect of Session for the “life satisfaction” (*F*(1, 27) = 71.72; *p* < 0.001), suggesting an increase in the HEDE group, as well as STR group. The interaction Session × Group was not significant. For the Cognitive Failures Questionnaire (CFQ), both the main effect of Session (*F*(1, 27) = 26.22; *p* < 0.001) and the interaction Session × Group (*F*(1, 27) = 6.78; *p* = 0.015) were significant. The interaction could be attributed to a substantial score reduction in the STR group after training, while the HEDE group showed a trend toward a decrease after the intervention. For the “emotional exhaustion” scale (MBI), the analysis revealed no interaction but a main effect of Session (*F*(1, 27) = 16.36; *p* < 0.001), suggesting a reduction in exhaustion in both groups. For the “demands” scale of the (PSQ), the analysis revealed an effect of Session (*F*(1, 27) = 16.23; *p* < 0.001) and an interaction Session × Group (*F*(1, 27) = 4.22; *p* = 0.048) that was due to a reduction of experienced demands in the STR group. Furthermore, there was an effect for the “tension” scale (PSQ) (*F*(1, 27) = 23.57; *p* < 0.001), indicating reduction of tension in both groups. Again, the interaction was not significant. For the scale “joy” (PSQ), there was an effect of Session (*F*(1, 27) = 7.89; *p* = 0.009) due to an increase in perceived enjoyment in the HEDE group whereas the increase in the STR group after training was not significant. No interaction was found. For the analysis of the scale “perceived worry” (PSQ) there was an interaction Session × Group due to a stronger reduction in STR than the HEDE group. Finally, regarding the total score of the (PSQ), the analysis yielded an effect of Session (*F*(1, 27) = 27.43; *p* < 0.001), and an interaction Session × Group (*F*(1, 27) = 6.26; *p* = 0.019) due to substantial reduction of the total PSQ-score in the STR group compared to the HEDE group.

**Table 8:** Training effects in stress measuring questionnaires in Sample 1.

Dimension	Group	Difference T3-T2	ANOVA between the Groups			ANOVA within the Groups			
		*ΔM (SD)*	*F*	*Df*	*p*	*F*	*df*	*p*	*d*
*Work-related behavior and experience pattern (AVEM)*									
Distancing ability	STR HEDE	0.93 (2.06) 0.73 (2.89)	< 1	1.27	n.s.	2.857 < 1	1.13 1.14	0.115 n.s.	−0.31
Inner peace and balance	STR HEDE	−0.57 (1.60) 1.27 (1.79)	**8.430**	**1.27**	**0.007**	1.778 **7.499**	1.13 **1.14**	0.205 **0.016**	0.19 −0.36
Life satisfaction	STR HEDE	3.15 (1.99) 2.94 (1.87)	< 1	1.27	n.s.	**34.762** **36.926**	**1.13** **1.14**	**<0.001** **<0.001**	−1.50 −0.97
Offensive problem solving	STR HEDE	0.57 (3.11) 0.13 (1.96)	< 1	1.27	n.s.	< 1 < 1	1.13 1.14	n.s. n.s.	
Striving for perfection	STR HEDE	−0.36 (3.18) −0.53 (1.25)	< 1	1.27	n.s.	< 1 2.748	1.13 1.14	n.s. 0.120	0.16
*Cognitive Failures Questionnaire (CFQ)*									
Total score	STR HEDE	−7.57 (5.96) −2.47 (4.54)	**6.783**	**1.27**	**0.015**	**22.611** 4.410	**1.13** 1.14	**<0.001** 0.054	0.57 0.24
*Maslach Burnout Inventory (MBI)*									
Emotional exhaustion	STR HEDE	−0.66 (0.81) −0.43 (0.64)	< 1	1.27	n.s.	**9.451** **6.684**	**1.13** **1.14**	**0.009** **0.022**	0.61 0.40
*Perceived Stress Questionnaire (PSQ)*									
Demands	STR HEDE	−15.24 (15.12) −4.89 (11.68)	**4.292**	**1.27**	**0.048**	**14.222** 2.630	**1.13** 1.14	**0.002** 0.127	0.84 0.56
Tension	STR HEDE	−17.62 (19.85) −13.12 (13.93)	< 1	1.27	n.s.	**11.033** **13.310**	**1.13** **1.14**	**0.006** **0.003**	0.84 0.64
Joy	STR HEDE	7.62 (19.37) 10.22 (14.66)	< 1	1.27	n.s.	2.167 **7.289**	1.13 **1.14**	0.165 **0.017**	−0.37 −0.41
Worries	STR HEDE	−14.76 (11.75) −5.33 (6.76)	**7.142**	**1.27**	**0.013**	**22.112** **9.333**	**1.13** **1.14**	**<0.001** **0.009**	0.93 0.24
Total score	STR HEDE	−0.67 (0.51) −0.24 (0.42)	**6.261**	**1.27**	**0.019**	**24.088** **4.772**	**1.13** **1.14**	**<0.001** **0.046**	1.03 0.49

Differences from post- and pre-measurement in stress-related questionnaires for the STR (*N* = 14) and HEDE (*N* = 15) groups in Sample 1. The differences (T3 – T2) were compared between the groups and the values at T2 and T3 were compared within groups. STR = Stress management training; HEDE = psychological health promotion training; *M* = mean; *SD* = Standard Deviation; *F* = F-value; *df* = degree of freedom; *p* = significance; *d* = effect size. Significant values are printed in bold.

Table 9 summarizes the effects of the stress measuring questionnaires in Sample 2. The analysis of the “life satisfaction” (AVEM) revealed an interaction Session × Group (*F*(1, 29) = 14.01; *p* < 0.001) and a main effect of Session (*F*(1, 29) = 108.76; *p* < 0.001). Within-group analyses revealed a stronger increase in the STR than in the HEDE group after training. No further effects or interactions were found for the other scales of AVEM. For the CFQ, there was an effect of Session (*F*(1, 29) = 11.10; *p* = 0.002), but the interaction Session × Group was not significant. Within-group analyses revealed an improvement in the STR group, and a trend toward an improvement in the HEDE group. For the “demands” scale (PSQ), the analysis yielded an effect of Session (*F*(1, 29) = 4.22; *p* = 0.049), while the interaction was not significant. The analyses within the groups showed reduction in experienced demands in the STR group only. For the scale “joy” (PSQ), there was an interaction Session × Group that was due to an increase in perceived joy from after training in the STR group but not in the HEDE group. For the “perceived worry” scale (PSQ), the interaction Session × Group was significant. However, there were only trends toward an increase in worry in the HEDE group and a decrease in the STR group. The main effect of the Session was not significant.

**Table 9:** Training effects in stress measuring questionnaires in Sample 2.

Dimension	Group	Difference T3-T2	ANOVA between the Groups			ANOVA within the Groups			
		*ΔM (SD)*	*F*	*Df*	*p*	*F*	*df*	*p*	*d*
*Work-related behavior and experience pattern (AVEM)*									
Distancing ability	STR HEDE	0.85 (1.26) −0.08 (2.34)	1.935	1.29	0.175	**7.318** < 1	**1.15** 1.14	**0.016** n.s.	−0.31
Inner peace and Balance	STR HEDE	1.38 (2.78) 0.13 (2.69)	1.593	1.29	0.217	3.920 < 1	1.15 1.14	0.066 n.s.	−0.53
Life satisfaction	STR HEDE	4.81 (1.97) 2.27 (1.79)	**14.018**	**1.29**	**0.001**	**95.118** **23.989**	**1.15** **1.14**	**<0.001** **<0.001**	−2.75 −0.95
Offensive problem solving	STR HEDE	−0.69 (2.87) −0.17 (2.55)	< 1	1.29	n.s.	< 1 < 1	1.15 1.14	n.s. n.s.	
Striving for perfection	STR HEDE	0.25 (3.38) −0.48 (2.16)	< 1	1.29	n.s.	< 1 < 1	1.15 1.14	n.s. n.s.	
*Cognitive Failures Questionnaire (CFQ)*									
Total score	STR HEDE	−6.07 (8.99) −2.73 (5.04)	1.598	1.29	0.216	**7.299** 4.420	**1.15** 1.14	**0.016** 0.054	0.48 0.37
*Maslach Burnout Inventory (MBI)*									
Emotional exhaustion	STR HEDE	−0.11 (1.07) −0.07 (0.48)	< 1	1.29	n.s.	< 1 < 1	1.15 1.14	n.s. n.s.	
*Perceived Stress Questionnaire (PSQ)*									
Demands	STR HEDE	−10.42 (18.69) −3.56 (19.17)	1.018	1.29	0.321	**4.968** < 1	**1.15** 1.14	**0.042** n.s.	0.51
Tension	STR HEDE	−5.00 (17.29) 0.89 (19.98)	< 1	1.29	n.s.	1.337 < 1	1.15 1.14	0.266 n.s.	0.25
Joy	STR HEDE	7.50 (13.31) −4.44 (16.07)	**5.108**	**1.29**	**0.031**	**5.084** 1.148	**1.15** 1.14	**0.04** 0.302	−0.40 0.22
Worries	STR HEDE	−8.33 (23.16) 5.78 (11.23)	**4.557**	**1.29**	**0.041**	2.072 3.970	1.15 1.14	0.171 0.066	0.38 −0.28
Total score	STR HEDE	−0.27 (0.65) −0.02 (0.64)	1.161	1.29	0.290	2.818 < 1	1.15 1.14	0.114 n.s.	0.38

Differences from post- and pre-measurement in stress-related questionnaires for the STR (*N* = 16) and HEDE (*N* = 15) groups in Sample 1. The differences (T3 – T2) were compared between the groups and the values at T2 and T3 were compared within groups. STR = Stress management training; HEDE = psychological health promotion training; *M* = mean; *SD* = Standard Deviation; *F* = *F*-value; *df* = degree of freedom; *p* = significance; *d* = effect size. Significant values are printed in bold.

In summary, particularly in Sample 1 clear changes were evident in both stress management groups after the interventions, with partially high effect sizes. In both training groups, scores improved in perceived “life satisfaction” (AVEM), “emotional exhaustion” (MBI), and on the scales “tension,” “worry,” in everyday cognitive failures (CFQ), and the total score of the Perceived Stress Questionnaire (PSQ). In the HEDE group there were positive changes for the scales “Inner calm and balance” (AVEM) and “happiness” (PSQ). The STR group improved their scores in the perceived “demands” (PSQ). In Sample 2 the effects have not be confirmed to the same extent, as the analysis of the HEDE group only showed an improvement in the “life satisfaction” scale (AVEM). The STR group also showed fewer positive changes than in the Sample 1. The effect sizes determined were in the moderate to large range for the “life satisfaction” scale (AVEM) with *d* = −2.75 (STR) and *d* = −0.95 (HEDE).

There were no significant interactions Session × Group in each of the cognitive tests, suggesting that different types of stress-management training did not differently influence the effects of cognitive training.

### Stress-related measures at baseline

The data presented above showed effects of training in stress-related parameters for Samples 1 and 2. Next, the samples were compared with respect to the stress-related variables at the baseline (T1). For this purpose, analyses were performed with the factor Sample (Sample 1 vs. 2). The results are shown in Table 10.

**Table 10:** Comparison between Sample 1 (*N* = 29) and Sample 2 (*N* = 26) regarding stress-related variables at baseline (T1).

Variable	Sample 1	Sample 2	ANOVA between groups		
	*M (SD)*	*M (SD)*	*F*	*df*	*p*
*Work-related behavior and experience pattern (AVEM)*					
Distancing ability	14.10 (3.47)	14.85 (3.34)	<1	1.53	n.s.
Inner peace and balance	12.59 (2.86)	14.35 (2.19)	**6.450**	**1.53**	**0.014**
Life satisfaction	10.45 (2.03)	14.38 (2.61)	**39.457**	**1.53**	**<0.001**
Offensive problem solving	13.28 (3.47)	14.46 (3.30)	1.674	1.53	0.201
Striving for perfection	14.21 (3.34)	11.88 (2.19)	**9.041**	**1.53**	**0.004**
*Cognitive Failures Questionnaire (CFQ)*					
Total score	59.89 (8.87)	53.92 (9.81)	5.612	1.53	**0.022**
*Maslach Burnout Inventory (MBI)*					
Emotional exhaustion	2.85 (1.04)	2.49 (0.66)	2.267	1.53	0.138
*Perceived Stress Questionnaire (PSQ)*					
Demands	48.97 (21.18)	37.60 (20.42)	**4.086**	**1.53**	**0.048**
Tension	50.11 (18.31)	35.13 (20.09)	**8.376**	**1.53**	**0.006**
Joy	45.29 (17.49)	55.38 (18.06)	**4.429**	**1.53**	**0.040**
Worries	45.29 (19.87)	27.44 (18.69)	**11.696**	**1.53**	**0.001**
Total score	2.83 (0.71)	2.27 (0.79)	**7.635**	**1.53**	**0.008**

*M* = mean; *SD* = Standard Deviation; *F* = *F*-value; *df* = degree of freedom; *p* = significance. Significant values are printed in bold.

For the “work-related behavior and experience pattern” (AVEM), the “inner calm and balance” and “life satisfaction” scales the scores in the Sample 1 were lower than in Sample 2. Also, for the scale “life satisfaction” Sample 1 showed a substantially lower score than sample 2. For the “striving for perfection” Sample 1 scored higher than Sample 2.

Regarding cognitive failures in daily life (CFQ), Sample 1 scored higher than Sample 2.

Higher values in Sample 1 than in Sample 2 were also observed for all scales of the Perceived Stress Questionnaire (PSQ), that is on “demands,” experienced “tension,” and “worry.” In line with this, the scale “joy” was lower in Sample 1 than Sample 2. Consequently, for the total PSQ-score, Sample 1 showed higher values than Sample 2.

In summary, nine of the total twelve scales showed lower values in Sample 1 than in Sample 2. Three variables that did not reach significance, but also showed lower values numerically. Even though the different levels of stress in the two training samples cannot be attributed causally to the different tense situation in the factory, the results indicate a different sense of well-being and stress experience in the two samples.

### Changes of the cortisol awakening response after stress management training

Expectedly, there was an effect of Saliva Measure (*F*(1, 27) = 33.02; *p* < 0.001), showing the usually observed increase of cortisol (from *M* = 4.42 ng/mL after awaking to *M* = 8.14 ng/mL 30 min later). In Sample 1, there was an effect of Session (*F*(1, 27) = 7.39; *p* = 0.011), showing a reduction of cortisol release from *M* = 7.07 ng/mL at T2 to *M* = 5.5 ng/mL at T3. No effect of STR vs. HEDE group was found (*F* < 1). More importantly, there was an interaction Session × Group (*F*(1, 27) = 5.44; *p* = 0.027), suggesting a reduction of CAR in the STR group from *M* = 7.74 to *M* = 4.82 ng/mL (*F*(1, 27) = 12.40; *p* = 0.004). This effect was mainly due to reduction of the second saliva measure from *M* = 10.85 at T2 to *M* = 6.04 ng/mL at T3, whereas no changes were observed in the saliva concentrations immediately after awaking (*M* = 4.63 ng/mL at T2 to *M* = 3.60 ng/mL at T3), resulting in an trend for the interaction Session × Saliva Measure (*F*(1, 27) = 4.43; *p* = 0.055). No changes in cortisol release between T2 (*M* = 6.39 ng/mL) and T3 (*M* = 6.17 ng/mL) were found in the HEDE group (*F* < 1).

**Figure 2:**
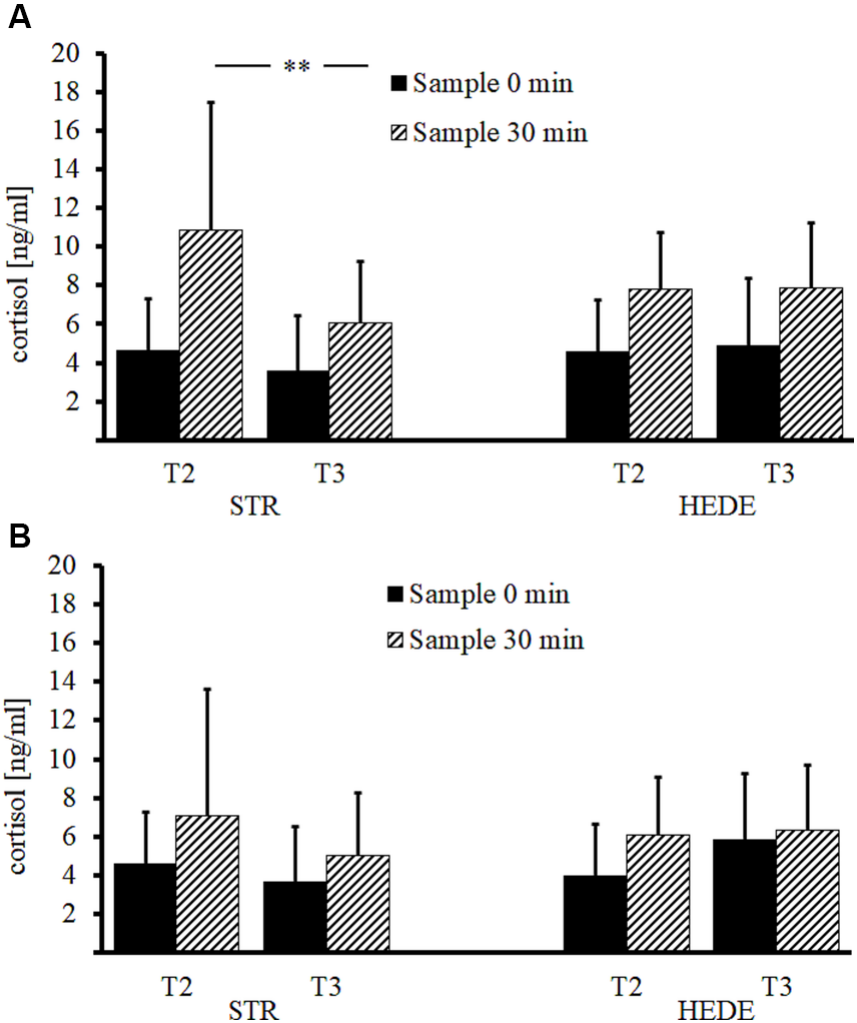
Results of the CAR in the STR and HEDE groups and both study samples. The CAR results are shown separately for both saliva measures immediately after awaking (saliva sample 0) and 30 min after awaking (saliva sample 30) for Sessions T2 and T3 and Study Sample 1 **(A)** and Sample 2 **(B)**.

In Sample 2, there was no main effect or Session or Group (both *F*’s < 1). Again, there was an effect of Saliva Measure with higher cortisol concentration 30 min after awaking (*M* = 6.03 vs. *M* = 4.57 ng/mL); (*F*(1, 24) = 14.70; *p* < 0.001). Moreover, there was a trend for an interaction Session × Group (*F*(1, 24) = 3.81; *p* = 0.062), indicating a similar pattern as in Sample 1, but the differences between Sessions were not substantial either in the STR (*F*(1, 24) = 2.21; *p* = 0.157) or HEDE group (*F*(1, 24) = 2.29; *p* = 0.164). No further effects or interactions were found. The results for the Sample 1 and 2 are shown separately in Figure 2.

Finally, to document differences in the level of cortisol release between Sample 1 and Sample 2, an ANOVA with the factors Saliva Measure and Sample at baseline (T1) was conducted. Indeed, the results showed an effect of Sample (1 vs. 2) with higher cortisol concentrations in Sample 1 than Sample 2 (*M* = 7.47 vs. *M* = 5.19 ng/mL); (*F*(1, 56) = 6.10; *p* = 0.017), and an interaction Sample × Saliva Measure (*F*(1, 56) = 5.45; *p* = 0.023), suggesting that this difference was driven by the saliva measure 30 min after awaking (Sample 1: *M* = 9.97 ng/mL vs. Sample 2: *M* = 5.84 ng/mL), while the cortisol concentration immediately after awaking did not differ between Sample 1 and Sample 2 (*M* = 4.96 ng/mL vs. *M* = 4.55 ng/mL). In summary, the general level of CAR at baseline was higher in Sample 1 than in 2 according to the level of subjectively reported stress and corroborating the assumption that participants of the Sample 1 suffered from the tense situation in the factory more than the Sample 2 did. While the HEDE training showed no effects on CAR there was an effect of the stress management training (STR) on CAR across both samples. In Sample 1 the reduction in CAR was significant and tended to be significant in Sample 2.

## Discussion

The main objective of the present study was to test the effectiveness of a cognitive and stress reducing intervention for middle-aged and older employees with mentally low-demanding and physically high-demanding, repetitive work. To evaluate the effectiveness of the cognitive intervention, psychometric tests were used at three time points to track changes in the crucial cognitive domains. Participants were divided into two groups: the training group took part in a cognitive training program over a period of three months and was retested in a follow-up measurement three months after the end of the intervention to assess the sustainability of the training effects. Participants in the wait list control group received at the beginning of the training phase eight sessions of either a stress management training (STR) or a psychological health promotion training (HEDE) which was followed by a shortened cognitive training. The combined training for the wait list control group started after completion of the cognitive intervention of the regular training group. This approach was used in two independent samples with one year delay. The results of the interventions are summarized in Table 11.

**Table 11:** Summary of the effects of cognitive and stress-management interventions in Sample 1 and 2.

		*Sample 1*	*Sample 2*
		*Cognitive effects*	
COG	T1 vs. T2	Digit-Symbol ↑ VLMT Recognition ↑	LPS-6 ↑ Digit-Span Forward ↑ TAP ↑ TMT-B ↑ TMT B-A ↗
	T2 vs. T3	D2 concentration ↑ D2 correct symbols ↑ Digit-Symbol ↑ TAP ↑ Stroop ↗ LPS-3 ↑ VLMT recognition ↓	LPS-7 ↓
		*Stress management effects*	
STR	T2 vs. T3	AVEM: Life satisfaction ↑ CFQ ↑ MBI ↑ PSQ: Demands ↑ PSQ: Tension ↑ PSQ: Worries ↑ PSQ: total ↑	AVEM: Distracting ability ↑ AVEM: Life satisfaction ↑ AVEM: Total score ↑
HEDE	T2 vs. T3	GHQ total ↑ GHQ: Physical well-being ↑ GHQ: Task management in the partnership ↑ GHQ: Coping with tasks at work ↑ AVEM: Inner peace and balance ↑ Life satisfaction ↑ CFQ ↗ MBI ↑ PSQ: Tension ↑ PSQ: Joy ↑ PSQ: Worries ↑ PSQ: total ↑	GHQ total ↗ AVEM: Life satisfaction ↑ PSQ: Demands ↑ PSQ: Joy ↑

**↑** indicates significant improvements, **↗** indicates a trend for improvement, **↓** indicates significant deterioration.

### Effects of cognitive interventions

Generally, the study revealed several positive outcomes, indicating that a short, trainer-guided multidimensional cognitive training is efficient to enhance performance in fluid cognitive functions in middle-aged and older employees with long history of repetitive work. However, the samples showed different results: whereas Sample 1 showed no training effects immediately after the training, such effects were seen 3 months later. This may suggest a slower accumulation of training-related changes, presumably due to a lower motivation to engage in the training, which could well be related to the strained economic situation of the company and the associated fears for the professional existence of the employees. In contrast, Sample 2, measured during a time when the occupational perspective was largely stabilized, showed improvements directly after the training in most of the cognitive functions, while no further enhancement was observed at follow-up (i.e., ceiling effect). This demonstrates stability of the achieved training effects, at least over a period of 3 months. The magnitude of the effect sizes is comparable to effect sizes in other intervention studies (Ball et al., 2002; Craik and Bialystok, 2006; Klusmann et al., 2010; Strobach and Karbach, 2021). Overall, the training effects and effect sizes obtained clearly support for the use of such cognitive interventions, as cognitive abilities have been substantially improved which are relevant to everyday life but known to be susceptible to age-related decline already early in adult life (Craik and Bialystok, 2006; Raz and Rodrigue, 2006). There were no significant changes in the wait list control groups in both samples, supporting the view that the observed effects are causally due to the intervention and not to repeated tests.

Training-related improvements were observed in information processing speed, word fluency, divided attention, verbal short-term and working memory, visual search, and task switching, as summarized in Table 11. These cognitive improvements were also found in the group that received combined (STR/HEDE + COG) training, even though the cognitive training was much shorter (12 instead of 20 sessions). This shows that even a relatively brief cognitive training is accompanied by a clear improvement in some fluid cognitive functions, which is in line with other training studies (Lampit et al., 2014; Hardy et al., 2015; Shawn Green et al., 2019; Strobach and Karbach, 2021). The functions that showed improvements were work-relevant and included crucial executive functions. Moreover, we observed far-transfer to daily activities assessed by the Cognitive Failures Questionnaire after the combined intervention, relative to the purely cognitive training. This may be presumably due to the stress intervention in the group that received the combined training, as outlined below.

In addition to the general effects, differential training effects were also analyzed. The subgroups formed for this purpose differed in their age, cognitive baseline level (low/high) as well as in their shift type (permanent night shift/regular change from early and late shift). Differences in training effects were only found in auditory–visual abilities (TAP) and logical thinking suggesting lower training-related gains in younger night shift worker in Sample 1 and in older night shift workers in Sample 2. However, the differential effects were rather marginal. The lack of substantial differential training effects showed that cognitive training has benefited all participants, regardless of age, cognitive baseline level, or shift work.

Finally, it is remarkable that the present results point to age-related differences in the initial cognitive baseline levels, although the sample of participants had a lower average age (47 years) and a lower age-span (40–57 years) than in most other aging studies (Hultsch et al., 2002; Hedden and Gabrieli, 2004). This clearly supports the need of early promotion of these age-sensitive domain, that is, at an age when cognitive training does not seem necessary, at least in a cognitively healthy population.

### Effects of stress management interventions

The time gained by shortening the cognitive training was filled by two variants of stress management and psychological health education programs (STR vs. HEDE). To test the effectiveness of the two interventions, standardized questionnaires were completed on the stress- and health-related experience before and after the trainings. Moreover, as a physiological measure of the response to stressors, the level of the stress hormone cortisol in saliva was measured (CAR) (Adam et al., 2006; Stalder et al., 2016).

The results of the stress-reducing interventions showed enhancement in stress resilience. The perception of psychosocial stress (measured by PSQ-20) as well as the emotional exhaustion (measured by MBI) were lower after both variants of stress management training than before (Flaxman and Bond, 2010; Lloyd et al., 2017). Moreover, the scores of life satisfaction (measured by AVEM) were higher. In contrast, after purely cognitive training, there were no changes in these variables. This demonstrates a selective influence of both types of training on stress resilience, but stronger gains of the stress management training (STR) that was focused explicitly on stress-relevant cognitive aspects and stress-reducing methods such as progressive muscle relaxation. For example, there was only a slight (non-significant) decrease of failures in everyday life after the HEDE (assessed by the CFQ), whereas the effect was substantial after STR training. This suggests that stress management training with relaxation is more efficient in reducing absent-mindedness and slips of action than cognitive training or psychological health program alone. This was in line with Broadbent et al. (1982), who suggested a relation between CFQ score and stress resilience.

The findings show enhancement in subjective stress management measures in both training groups, with improvements in the STR training being more pronounced. Moreover, the reduction in cortisol awakening response in the morning of testing was only observed in the STR group. This indicates that stress management training, supported by the progressive muscle relaxation, decreased the physiological response to the expected daily stress (Adam et al., 2006; Nicolson et al., 2020).

### Differences between the samples

The differences in effectiveness of the cognitive training revealed an imbalance between Sample 1 and Sample 2 in favor of Sample 2. It can be assumed that this is related to the professional situation in which the groups were during the training: There was a different amount of short-time working days due to the tense situation in the factory, with participants of Sample 1 (21 days on average) being much more affected than Sample 2 (11 days). In addition to the associated loss of income, the increased fear of losing one’s job plays a particularly important role. The difference between samples was not only apparent in the reported stress perception, the level of cortisol at baseline was also substantially higher in Sample 1 than 2. It is well known that chronic stress associated with high cortisol level negatively influences learning capacity and disrupts some cognitive functions such as memory (Lupien et al., 2007, 2009; Marin et al., 2011). For the cognitive plasticity and learning capacity, individual motivation and the accompanying circumstances are of great importance (Greenwood and Parasuraman, 2010; Sterlemann et al., 2010; Schapkin et al., 2012; Fissler et al., 2013). Thus, it can be assumed that uncertainty and dissatisfaction about one’s own professional future produces chronic stress and had an unfavorable effect on motivation, emotion, and the efficacy of the cognitive training, and thus weakened the effects achieved by the training which occurred after a delay of 3 months. A further possibility is that after the acute stress period the formerly stressed group might have trained without being told so using freely available programs. This might have improved cognition in this group between T2 and T3. It is plausible to assume that both aspects played a role in the late training effect.

### Promotion of study sustainability

An important part of the study was to sensitize policymakers, employers, and occupational safety and health personnel in Germany to this topic and to emphasize the need for lifelong learning in occupational context.

As a first step, guidelines for cognitive training and stress management training were developed for trainers. This should enable health actors and interested users with appropriate prior knowledge to hold workshops or to train other employees as multipliers (train the trainer). For each training unit, the required materials, the contents to be conveyed, time specifications and further information have been summarized. In a second step, a two-day workshop was held with policymakers, employers, and occupational safety and health personnel from different occupational areas and insurances. In addition to teaching the theoretical basics and conducting the training, the agenda included the optimization of the practical suitability of the training concept through an exchange of experience. The results of the workshop were incorporated into the optimization of the guidelines (Freude et al., 2012, 2013). Further practical recommendations for occupational safety and health were summarized in Gajewski et al. (2023).

### Limitations

There is a methodological limitation of the study that needs to be acknowledged. We did not create a full experimental design with several groups that would be trained at the same time. Instead, a wait-list design was implemented with a combined intervention after the role of the passive control group was finished. Therefore, a direct comparison of training effects between the pure cognitive training conducted after the baseline intervention and the combined cognitive and stress management training conducted later was not possible. A full design would include 4 groups with a cognitive training, a stress-management intervention, and a combination of both, as well as a passive control group. Unfortunately, this could not be realized in the occupational context due to a limited number of volunteers and a small number of participants per group. In addition, the comprehensive measurements in the control group without any intervention would be disappointing for the participants who were interested in taking part in a training study. The combination of cognitive and stress management training was additionally intended to compensate for the long passive phase without intervention.

### Conclusions and future directions

The described comprehensive study conducted in an industrial setting showed that age- and work-related cognitive impairment and a high level of psychosocial stress in employees with long-term repetitive work can be substantially reduced by short cognitive and stress management trainings. This shows that cognitive and emotional functions can be improved even after decades of mentally unchallenging work and in a critical occupational situation. Neuronal plasticity that persists even in old age is of crucial importance here (Greenwood and Parasuraman, 2010; Falkenstein and Gajewski, 2021). However, the learning capacity under chronic stress is apparently reduced and can be restored by short stress management und relaxation training that may even led to enhancement of attention and memory in everyday life. Thus, to attenuate cognitive decline of the aging workforce, some simple methods such as combined cognitive and stress management trainings can be used, which ideally should be offered as short units during breaks or even work hours and supported by the employer and occupational safety and health personnel. Because trainer-guided trainings are time consuming, expensive, and difficult to organize in an occupational context, interactive systems that recommend different human-centered training units could reflect an economic solution. Indeed, a follow-up project funded by the EU Commission sustAge, sustainable work through technology-assisted enhancement of cognitive abilities of older employees (Athanassiou et al., 2021, www.sustAGE.eu) aimed at developing a smart system supporting cognitive, emotional, and physical constitution of older adult workers in manufacturing. This interactive system is intended to detect stress, fatigue or impaired performance using sensor- and voice-recognition algorithms, and to recommend alternative work activities (e.g., workplace rotation) or micro breaks during which some units of cognitive training or stress reducing units can be performed. Such a solution would enhance occupational safety and health, increase cognitive and emotional functioning, job satisfaction and productivity in older workforce which may prevent premature retirement.

## Data availability statement

The data presented in the study are deposited in the Figshare repository, doi: 10.6084/m9.figshare.24016827.

## Ethics statement

The study was reviewed and approved by the local Ethics Committee of the Leibniz Association at IfADo in accordance with the declaration of Helsinki. All participants were informed about the purpose of the study and gave written informed consent.

## Author contributions

PG contributed to conceptualization, methodology, investigation, data curation, supervision, writing-original draft, and project administration. CS contributed to formal analysis, conceptualization, investigation, data curation, and writing-original draft. JZ contributed to conceptualization, funding acquisition, project administration, and supervision. EW provided resources. SG contributed to methodology, writing-original draft, and editing. MF contributed to conceptualization, methodology, investigation, supervision, funding acquisition, project administration, writing-original draft, and editing. All authors contributed to the article and approved the submitted version.

## Funding

The research reported in the present article was conducted within the PFIFF2-Project (program for improving cognitive abilities in older employees) in the framework of INQA – New Quality of Work which was funded by Federal Ministry of Labour and Social Affairs (BMAS), Germany.

## Conflict of interest

MF is head of the Institute for Working Learning Ageing (ALA), Bochum, Germany.

The remaining authors declare that the research was conducted in the absence of any commercial or financial relationships that could be construed as a potential conflict of interest.

## Publisher’s note

All claims expressed in this article are solely those of the authors and do not necessarily represent those of their affiliated organizations, or those of the publisher, the editors and the reviewers. Any product that may be evaluated in this article, or claim that may be made by its manufacturer, is not guaranteed or endorsed by the publisher.
