# Supplementary material for: Effects of cognitive and stress management training in middle-aged and older industrial workers in different socioeconomic settings: a randomized controlled study
Source: Front Psychol. 2023 Sep 12;14:1229503. doi: 10.3389/fpsyg.2023.1229503 (PMC10523316; doi:10.3389/fpsyg.2023.1229503)
Supplement: Supplementary file 1 [file Data_Sheet_1.pdf]

Supplementary Material

Supplementary Methods

Cognitive interventions

In the first training phase, mental activation training (MAT; Lehrl et al. 1994, www.gfg-online.de, Sattler-Rommel 2005) and other paper-based exercises (Sudoku etc.) were trained. PC-based exercises from the programs FreshMinder2 (www.freshminder.de), USM Brain Trainer (www.usm.de), Ahano-PEDS3 (www.ahano.de) and Happy Neuron (www.happyneuron.de) were later used. Additionally, a PC-based task switching training was included (adapted from Karbach & Kray, 2009). Please refer to the Appendix (Table A 1) in Gajewski & Falkenstein, 2012 for further details of the training program.

Stress management training

The Progressive Muscle Relaxation (PMR; Jacobson, 2006). The technique involves learning to monitor the tension in specific muscle groups. This tension is then released, as attention is directed towards the differences felt during tension and relaxation. The tension of the muscle parts is held for about five to seven seconds and released with the exhalation, which leads to the relaxation of the respective muscle region. This state of relaxation is held for 30-45 seconds. During this process the participant focuses on the relaxation in the respective muscle areas. The participants were instructed to such an extent that they were able to perform the PMR exercises on their own and integrate them into their daily lives after completing the training.

Psychological health promotion program (HEDE training®; Franke & Witte 2009), was based on the concept of salutogenesis according to Antonovsky (1987). In contrast to the pathogenetic view, which deals with the causes of disease, the salutogenetic model examines the question of why people - despite stress and health-threatening stimuli - remain healthy. The states of health and disease are viewed in the salutogenetic and are seen as the respective end points of a continuum. The individual position between "health" and "illness" may change in the course of life. The assumption is that no one is ever completely healthy, but also never completely ill (Mittelmark et al., 2021). Antonovsky postulates "generalized resistance resources" as the factors that contribute decisively to a constructive handling of stressors. As a superordinate personal resource, he postulated the "sense of coherence", which encompasses the elements of comprehensibility, manageability, and significance. The HEDE-Training® aims at strengthening the feeling of coherence - both through the expansion of the repertoire of coping behaviour and through the development of resource-promoting experiences and behaviour (Franke & Witte 2009).

Psychometric tests

d2 Test

The attentional endurance test d2 (Brickenkamp, 2002) requires fast search for stimuli according to predefined visual features. It consists of 14 lines, each composed of 47 characters (the letters "d" and "p" with one to four dashes (‘) above and/or below each letter). The subjects have 20 seconds per line to cross out the target stimuli - "d" with two dashes. The total number of correctly identified d’s with two dashes represents the test score. The d2 test is a measure of focused and sustained attention as well as processing speed.

The concentration performance and the speed (number of target objects processed) are measured with a high degree of accuracy (Cronbach's alpha between .89 and .95 depending on the age group, retest reliability after 1 and 10 days = .94 and .91 and .85 and .92 respectively). The characteristic value for accuracy (error rate) has satisfactory reliabilities (Cronbach's alpha: .80 to .91, retest reliability: .84 and .47).

LPS

The performance testing system (LPS), consisting of 14 subtests, was developed by Horn (1983) and measures both fluid and crystallized aspects of intelligence. It has been normed on a large sample and is a reliable and valid measurement of intelligence. Each subtest has an increasing difficulty. For the present study the following subtests were used:

In the LPS-1, measures crystalline intelligence operationalized in the form of a verbal factor. In this task, the participant is presented with a list of words, each of which has a spelling error that must be detected and crossed out. Maximum of 40 errors can be detected in 2 minutes. Dependent variable is the number of correctly detected spelling errors.

The LPS-3 measures logical reasoning, i.e., the ability to think logically as an aspect of fluid intelligence. The aim is to indicate the incongruent element in each row of eight logically arranged symbols. The highest score to be achieved is 40. The respondent was given five minutes to complete the test.

The LPS-6 measures word fluency and cognitive flexibility as index of fluid intelligence. In this test, participant is asked to identify as many words as possible from the three given initial letters. For each initial letter the participant was given one minute. The total number of all words written down (without repetition, without rule violation) were used as the test score in the evaluation.

The LPS-7 requires a mental rotation of letters in the plane – an ability attributed to fluid intelligence. The task consists of crossing out those symbols that are recognized as mirror images. The time limit for this subtest is two minutes. A maximum of 40 recognized symbols can be achieved. The number of correctly crossed out numbers or letters was used as dependent variable for the evaluation.

The split-half reliability of the overall LPS is .99. The parallel test reliability is .94. The retest reliability of the subtest groups for a period of 2 to 3 years between .67 and .85 and for the total battery at .87.

NAI

Nürnberg Age Inventory (NAI; Oswald & Fleischmann, 1999) can be used to examine basic cognitive performance, aspects of behavior, well-being of subjects of older age. It has been normed on a large sample and is a reliable and valid measurement of cognitive functions (Oswald & Fleischmann 1999). Norming samples and parallel forms are available.

Stroop test. The color-word interference test FWIT (Stroop 1935), consists of three tasks to test response inhibition to irrelevant stimuli. In the first task, color words printed in blank ink are to be read as quickly as possible (e.g., "red", "green"). The second task consists of naming the color bars. In the third task - the interference condition - the subjects are asked to name a series of color of color words in which a color word is printed in colors which did not match the names of the colors (e.g., “GREEN” was printed in red). Here, word meaning, and print color are incongruent, with inhibition performance consisting of naming the color rather than reading the word as an automated response. The processing time of the interference condition as well as the difference between the interference condition and the second task ("name colors") are dependent variables. The reliability

Digit-span (DS). In the first part of the task (digit-span forward; DS-F), series of digits with increasing length were orally given by the experimenter. The number of correctly memorized series of digits indicates short-term memory performance. Correct reproduction of two series with the same length indicate correct response. The maximal performance is nine numbers.

In the second part of the task (DS-B), digit-span backward, the presented number sequences must be repeated in reverse order. A maximum of eight consecutive numbers can be repeated. Two successive series of numbers with the same length indicate correct response. This version of the task assesses working memory capacity.

Digit-Symbol-Test (DST). The number-symbol test measures aspects of focused attention and psychomotor speed (Oswald & Fleischmann 1999). In this test, the symbols on the test sheet have to be matched to the numbers 1 to 9 within 90 seconds. The maximum score is 93. The number of correct number-symbol assignments served as dependent variable.

Reliability for the speed tests ranges from .74 to .98. Reliability for the memory tests ranges from .60 to .81. Reliability for the rating scales ranges from 73 to .93. Internal consistency ranges from .69 to 94. Inter-rater reliability ranges from .63 to .81.

Test Battery for Attention Assessment (TAP)

The computerized Test Battery for Attention (Version 2.1; Zimmermann & Fimm 2002) offers a comprehensive diagnosis of several types of attention. The divided attention subtest was used in the present work. It requires the subjects to simultaneously process a visual and an auditory task. In the visual task, the subject is asked to press a key whenever a square consisting of four crosses appears on the screen. In the acoustic task, the subject is presented with alternating high and low tones. In the acoustic task, the subject is presented with alternating high and low tones and is asked to respond by pressing a key when two identical tones are presented in succession. The time is three and a half minutes. In the statistical evaluation the reaction times (in ms) for the acoustic and the visual task as well as the total number of errors for both tasks together.

Reliability for the speed tests is .97. Reliability for the error rates ranges between .53 and .92.

Trail Making Test (TMT)

The Trail Making Test (Reitan, 1992) is a paper-pencil test consisting of two parts. Part A measures processing speed and short-term memory by asking the participants to connect the numbers 1 through 25 consistently in ascending order (Lüthi, 2009).

In part B, the letters A to L and the numbers 1 to 13 are to be alternately connected in ascending order. In this dual task parallel processing of the two different subtasks "numbers" and "letters" is

required. Part B measures switch ability and divided attention. The difference between processing times for part B and A measuring components of executive functions were included as dependent variable in the analysis.

Cronbach's alpha ranges between .70 and .74 for parts A and B, respectively and a composite index is .92 (Reynolds, 2002).

Verbal Learning and Memory Test (VLMT)

The VLMT (Helmstaedter et al., 2001) is a translation of the AVLT (Auditory Verbal Learning Test; Schmidt, 2016). The VLMT measures the performance of verbal declarative episodic memory, which plays a major role in the performance of everyday tasks (Schaefer & Bäckman 2007). The test material of the VLMT consists of the one hand of a learning list and an interference list with 15 words each, which are semantically independent of each other. On the other hand, there is a recognition list with all words of the learning and interference list as well as 20 additional words. First, the subjects are presented with the learning list orally by the experimenter five times. After each presentation (trial 1 to 5), the words must be reproduced. This is followed by a single presentation of the interference list (trial I), which also must be immediately reproduced. After this, the subjects are asked to reproduce the learning list presented at the beginning (trial 6). After about 30 minutes, which are used for the other tests, the learning list should be reproduce once again (trial 7). Finally, the recall list, consisting of a total of 50 words, is presented orally. Here the task is to recognize the 15 words of the learning list. Dependent variables were the total number of words from the learning list that the subject recalled (trial 1 to trial 5), loss of words reproduced from the original list after the interference list (trial 5 minus trial 6) and from delayed free reproduction (trial 5 minus trial 7), and the recognized words of the recognition list (minus errors). There are two parallel forms that were used for the test repetitions.

Cronbach's alpha ranges between .68 and .87 depending on the parameter.

Questionnaires

Work Ability Index

Work ability was assessed with the WAI, which considers the workers' self-assessed physical and mental capacity in relation to work requirements, health status, and the worker resources (Ilmarinen, 2009, 2019). The questionnaire consists of seven self-reported parts with higher scores indicating better adjustment: (WAI1) "subjective estimation of current work ability compared with lifetime best", with scores ranging from 0 to 10; (WAI2) "subjective work ability in relation to job demands", with scores ranging from 2 to 10; (WAI3) "number of current diseases diagnosed by a physician", with five categories ranging from 1 to 7; (WAI4) "subjective estimation of work impairment due to diseases", with six categories scores ranging from 1 to 6; (WAI5) "sick leave during the past year", with five categories ranging from 1 to 5; (WAI6) "own prognosis of work ability two years from now", with three categories ranging from 1 to 7; and (WAI7) "mental resources", with four categories ranging from 1 to 4. The total score is calculated by summing up scores of seven WAI-categories. WAI can range between 7 and 49 points (table 1) and is classified into the following categories: poor (7 – 27), moderate (28 – 36), good (37 – 43), and excellent (44 – 49) WA. The WAI questionnaire was used at the baseline testing only.

Cronbach's alpha ranges between .53 and .83 depending on the sample.

Sense of Coherence Questionnaire

The short version of the Sense of Coherence Questionnaire (Antonovsky, 1987, German translation Abel, et al., 1995) consisting of 13 items was used to test the effectiveness of the HEDE Training®. As a screening instrument, the SOC measures the construct "sense of coherence," which is central to Antonovsky's (1987) salutogenesis model is described as a dispositional coping resource, that is related to increasing resilience to stressors. The sense of coherence is represented by three dimensions, which are referred to as understandability, manageability, and meaningfulness. A person can cope with strains and stress without health losses if he or she has a high score in the dimension of the sense of coherence. The 13 items were answered on a seven-point response scale. Five items from the area "understandability", four items from the area "meaningfulness" and four items from the area "manageability" describe the SOC short scale. The reliability coefficient of the SOC-13 short scale can be rated as good at .85 (Cronbach's alpha). The high correlation of the SOC-13 short scale (r = .94) with the total score of the SOC-29 long version can be regarded as evidence of the validity of the short scale (Schumacher et al. 2000b). Reference values for the SOC short scale are available (Schumacher et al. 2000a). Completing the questionnaire takes about five minutes. For the evaluation of the short version, a global factor is used, which is calculated from the averaged raw sum value over all 13 items.

Cronbach's alpha ranges between.74 and .91 depending on the sample.

General Health Questionnaire (GHQ-12)

The original version of the General Health Questionnaire (Goldberg & Williams 1991) measures the risk for the development of a psychiatric disorder in a multidimensional way. The shortened version with twelve items (GHQ-12) used here measures psychological distress in the past two weeks on a four-point scale. The individual items are added up to a total value, whereby values of twelve or higher can be interpreted as signs of mental stress. The questionnaire takes about five minutes to complete. The GHQ-12 has proven to be a valid screening instrument, also in the German translation (Goldberg et al. 1997).

Cronbach's alpha ranges between.73 and .90 depending on the sample.

Questions on physical well-being

To assess current physical well-being, Franke and Witte (2009) developed eleven items that are included in the evaluation as a total value. High scores reflect a positive reflect a positive body feeling. Answering the questions takes about five minutes.

The questions on coping with daily tasks (Franke & Witte, 2009) are used to ascertain the extent to which to what extent the respondents feel able to cope with their daily tasks. The respondents answer respondents only answer items from those task areas that are personally relevant to them. Since the number of relevant task areas varies between individuals, the items are evaluated individually. High values stand for an improvement in task mastery (Franke & Witte 2009). Answering the questions takes a maximum of five minutes.

No information about internal consistency is available.

Work-Related Behavior and Experience Pattern (AVEM-44)

The questionnaire on work-related behavior and experience patterns (AVEM; Schaarschmidt & Fischer, 2008) was used to test the training effects in the occupational context. This is a standardized diagnostic procedure for recording behavior and experience in relation to work and occupational requirements and assessing them from the point of view of health-relevant aspects. Using the AVEM, personality-specific in the form of patterns in the confrontation with these requirements can be diagnosed. The use of the AVEM is particularly suitable for testing intervention-related effects. The long version has 66 items and the short form used in the present study consists of 44 items.

Schaarschmidt and Fischer (2008) emphasize that personality, and thus personality-specific behavior and experience play a decisive role in the development of health and that the connection between personality and health is conveyed in particular through professional work. On a five-point scale ("completely agree" to "do not agree at all"), the subjects are placed on the following eleven dimensions, each of which is represented by four items:

- Subjective importance of work (significance of work in personal life)
- Professional ambition (striving for career advancement and success)
- Willingness to expend effort (willingness to devote personal energy to the fulfillment of the work task)
- Striving for perfection (demand for quality and reliability of one's own work performance)
- Ability to distance oneself (ability to recover psychologically from work)
- Resignation tendency in case of failure (tendency to resign oneself to failure and to give up)
- Offensive problem solving (active and optimistic attitude towards challenges and problems that arise)
- Inner peace and balance (experience of psychological stability and inner balance)
- Experience of success at work (satisfaction with professional achievements)
- Life satisfaction (satisfaction with the entire life situation, also beyond work)
- Experience of social support (trust in the support of close people, feelings of social people, feelings of social security)

These dimensions can be grouped into the three superordinate domains of work engagement, psychological Resilience, and Work-related Emotions. Filling out the questionnaire in the paper and pencil version pencil version takes about ten minutes. The calculation of the scale values was based on the questionnaire manual (Schaarschmidt & Fischer 2008). To test the effectiveness of the Stress Intervention and the HEDE-Training®, the scales "Striving for Perfection", "Life Satisfaction", "Distancing ability," "Offensive problem solving," and "Inner calm and balance. calmness and balance" were analyzed.
Cronbach's alpha ranges between.79 and .87 depending on the sample.

Perceived Stress Questionnaire (PSQ-20)

The Perceived Stress Questionnaire (Levenstein et al. 1993) is used to assess the subjective perception, evaluation, and further processing of stressors. The perception of stress is one of the decisive factors for the course of different diseases and disorders. In the present study the German short version of Fliege et al. (2001) was used. PSQ-20 consists of four dimensions (worry, tension, pleasure and demands), each characterized by five items. According to Fliege et al. (2001), the scales worry, tension, and joy represent the internal stress response whereas the demands scale refers to the perception of external stressors. Specifically, the items in the Worry scale refer to the experience of

stress in the form of worries, fears about the future, and feelings of frustration. The "Tension" scale includes exhaustion, imbalance, and lack of physical relaxation. The "Joy" scale is composed exclusively of positively worded items that can be interpreted as the experience of joy. The "demands" scale consists of items that depict the perception of primarily external demands such as lack of time, deadline pressure or task load. On a four-point Likert scale ("almost never" to "most of the time"), the subjects rate the total of 20 statements. Completing the questionnaire takes about five minutes.

Cronbach's alpha ranges between.80 and .86 depending on the sample.

Maslach Burnout Inventory (MBI)

MBI (Maslach & Jackson 1981, 1986; Maslach et al. 1996) is a valid instrument for multidimensional assessment of burnout and represents the only instrument that captures all core dimensions of burnout. The dimensions are: "Emotional Exhaustion," "Reduced Efficiency," and "Depersonalization." These three dimensions are operationalized by three subscales composed of a total of 22 items. The Emotional Exhaustion subscale represents the central quality of burnout and the most obvious manifestation of this syndrome. As a stress component, this dimension is expressed in feelings of overexertion, as well as depletion of emotional and physical resources. "Depersonalization" is accompanied by a distancing from one's work, expressed in a negative and indifferent manner to aspects of work. Reduced "Personal Accomplishment", as a self-evaluative dimension, is related to feelings of incompetence and a lack of effectiveness in professional life. In the present study, only the scale "Emotional Exhaustion" of the German version according to Büssing and Perrar (1992) was used. On a six-point Likert scale (1 = "does not occur at all"; 6 = "occurs very often"), the study participants rate the nine statements with respect to the frequency of their occurrence.

Cronbach's alpha ranges between.82 and .88 depending on the sample.

Cognitive Failures Questionnaire (CFQ)

The Cognitive Failures Questionnaire (Broadbent et al., 1982) is a 26-item questionnaire used for self-assessment of failures in perception, memory, and motor functions. On a five-point Likert scale (4 = *very often*, 0 = *never*), subjects rate the frequency of their everyday inattentions. Broadbent et al. (1982) suggest that the instrument may capture a general deficit of cognitive control and that a high CFQ score is associated with reduced stress resistance. In contrast to the original version, whose questions pertained to the past six months in the present study, the subjects were asked to answer the questions with respect to the past four weeks to be able to relate possible changes to the intervention carried out. Since this procedure was chosen for all measurement time points (Sessions) and groups, an influence on validity and reliability is unlikely (Westerberg et al., 2007). It takes about five minutes to complete the questionnaire. The sum value of the answered items was included in the evaluation (maximum score 100). The CFQ was used to test the effectiveness of the cognitive intervention, since an improvement in cognitive performance should also be reflected in personal perception in everyday life. On the other hand, the questionnaire was used to test the effectiveness of the stress interventions, since Broadbent et al. (1982) found a relation between a high CFQ score and decreased stress resistance.

Cronbach's alpha ranges between .85 and .93 depending on the sample.

Supplementary Results

Examination of comparability between the training and the waiting control groups at T1.

Supplementary Table 1

Baseline performance in cognitive tests in Sample 1

**Variable**	**COG (*N* = 29)** *M (SD)*	**CTRL (*N* = 29)** *M (SD)*	***p***
**Attentional endurance test (d2)**			
Total number of symbols	416.72 (79.06)	430.90 (73.54)	n.s.
Total Number of correct symbols	383.86 (70.71)	403.00 (61.79)	n.s.
Total number of errors[1.2]	29.82 (21.62)	27.93 (20.84)	n.s.
Concentartion performance	142.03 (31.76)	151.90 (23.42)	n.s.
**Performance Testing System (LPS)**			
LPS-1	19.00 (6.05)	18.69 (5.37)	n.s.
LPS-3	23.52 (3.76)	24.59 (4.58)	n.s.
LPS-6	29.48(7.85)	31.07 (8.11)	n.s.
LPS-7	17.83 (5.29)	18.76 (5.51)	n.s.
**Nürnberg Age Inventory (NAI)**			
digit-span forward	6.48 (1.02)	6.59 (1.12)	n.s.
digit-span backward	4.83 (1.44)	5.03 (1.43)	n.s.
Digit-Symbol-Test	50.31 (6.18)	51.69 (8.99)	n.s.
Stroop (s)[2]	39.52 (7.08)	39.78 (8.53)	n.s.
Stroop difference (s)[2]	17.34 (5.30)	17.52 (5.98)	n.s.
**Test Battery for Attention Assessment (TAP)**			
Auditive condition (ms)[2]	595.37 (100.90)	580.83 (87.62)	n.s.
Visual condition (ms)[2]	849.93 (119.20)	874.07 (123.37)	n.s.
Total number of errors)[1.2]	2.85 (3.56)	1.55 (2.16)	n.s.
**Verbal Learning and Memory Test (VLMT)**			
Total number of words	43.69 (8.75)	49.00 (7.45)	.016
Immediate reproduction[2]	2.24 (1.77)	1.93 (1.44)	n.s.
Delayed reproduction[2]	2.21 (2.23)	1.72 (1.39)	n.s.

Recognition performnace	11.11 (3.34)	12.38 (2.61)	.061
**Trail Making Test (TMT)**			
Version A (s)[2]	32.86 (9.99)	30.07 (8.67)	n.s.
Version B (s)[2]	74.28 (19.44)	68.72 (23.87)	n.s.
Difference B - A (s)[2]	41.41 (18.15)	38.66 (20.04)	n.s.
**Cognitive Failures Questionnaire (CFQ)**			
Total score	59.89 (8.87)	62.28 (13.12)	n.s.

Notes. COG = cognitive training group; CTRL = Control group; *N* = sample size; *M* = mean; *SD* = standard deviation; *p* = significance level.[1] Mann-Whitney-U-Test. [2] Lower values represent higher performance

Supplementary Table 2

Baseline performance in cognitive tests in Sample 2

**Variable**	**COG (*N* = 29) *M (SD)***	**CTRL (*N* = 29) *M (SD)***	***p***
**Attentional endurance test (d2)**			
Total number of symbols	418.42 (70.59)	413.66 (68.06)	n.s.
Total Number of correct symbols	383.73 (67.39)	393.25 (69.51)	n.s.
Total number of errors[1.2]	34.69 (25.51)	20.09 (19.38)	.019
Concentartion performance	140.27 (33.26)	153.13 (34.98)	n.s.
**Performance Testing System (LPS)**			
LPS-1	17.54 (5.55)	19.31 (5.72)	n.s.
LPS-3	22.88 (4.40)	24.84 (4.72)	n.s.
LPS-6	29.50 (7.18)	31.50 (7.38)	n.s.
LPS-7	19.23 (6.33)	19.34 (6.45)	n.s.
**Nürnberg Age Inventory (NAI)**			
digit-span forward	6.23 (0.91)	6.47 (1.08)	n.s.
digit-span backward	5.12 (1.03)	4.94 (1.01)	n.s.
Digit-Symbol-Test	56.35 (8.62)	57.78 (11.33)	n.s.
Stroop (s)[2]	38.69 (8.89)	34.71 (8.02)	n.s.
Stroop difference (s)[2]	15.96 (7.28)	13.74 (5.79)	n.s.
**Test Battery for Attention Assessment (TAP)**			

Auditive condition (ms)[2]	593.27 (92.99)	556.22 (80.82)	n.s.
Visual condition (ms)[2]	830.60 (72.89)	825.25 (97.61)	n.s.
Total number of errors[1.2]	4.15 (4.88)	2.69 (4.48)	n.s.
**Verbal Learning and Memory Test (VLMT)**			
Total number of words	51.00 (6.24)	50.81 (6.76)	n.s.
Immediate reproduction[2]	1.50 (1.24)	1.16 (1.29)	n.s.
Delayed reproduction[2]	1.58 (1.27)	1.50 (1.67)	n.s.
Recognition performnace	12.73 (2.39)	12.72 (2.33)	n.s.
**Trail Making Test (TMT)**			
Version A (s)[2]	30.77 (7.38)	28.19 (10.36)	n.s.
Version B (s)[2]	76.85 (30.37)	69.84 (22.97)	n.s.
Difference B - A (s)[2]	46.08 (27.94)	41.66 (18.25)	n.s.
**Cognitive Failures Questionnaire (CFQ)**			
Total score	53.92 (9.81)	58.97 (11.67)	.084

Notes. COG = cognitive training group; CTRL = Control group; *N* = sample size; *M* = mean; *SD* = standard deviation; *p* = significance level.[1] Mann-Whitney-U-Test. [2] Lower values represent higher performance.

Supplementary Table 3

Descriptive statistics in the cognitive training group and the waiting control group at T1 and T2 in Sample 1

Variable	Group	T1 *M (SD)*	T2 *M (SD)*	Difference T2-T1 *ΔM (SD)*
**Attentional endurance test (d2)**				
Total number of symbols	COG	416.72 (79.06)	436.89 (91.56)	20.17 (55.37)
	CTRL	440.66 (81.26)	464.07 (63.36)	23.41 (52.93)
Total Number of correct symbols	COG	383.86 (70.70)	407.62 (88.18)	23.76 (54.85)
	CTRL	422.52 (73.12)	448.48 (58.24)	25.97 (47.89)
Total number of errors[1]	COG	32.86 (26.81)	29.28 (27.72)	-3.59 (10.79)
	CTRL	18.48 (14.56)	15.59 (15.52	-2.89 (12.72)
Concentartion performance	COG	142.03 (31.76)	151.55 (35.89)	9.52 (16.71)
	CTRL	164.17 (21.23)	179.62 (25.89)	15.45 (12.31)
**Performance Testing System (LPS)**				
LPS-1	COG	19.00 (6.05)	19.07 (5.57)	0.07 (4.31)

	CTRL	18.34 (5.63)	20.72 (6.18)	2.38 (2.79)
LPS-3	COG	23.52 (3.76)	26.14 (5.06)	2.62 (3.21)
	CTRL	27.14 (4.45)	28.69 (4.56)	1.55 (3.36)
LPS-6	COG	29.48 (7.85)	30.38 (8.72)	0.89 (6.39)
	CTRL	33.28 (8.28)	36.62 (8.68)	3.34 (4.94)
LPS-7	COG	17.83 (5.29)	21.97 (5.05)	4.14 (3.45)
	CTRL	21.34 (6.52)	20.83 (5.42)	-0.52 (4.79)
**Nürnberg Age Inventory (NAI)**				
digit-span forward	COG	6.48 (1.02)	6.38 (1.21)	-0.10 (1.05)
	CTRL	6.48 (1.27)	7.07 (1.41)	0.59 (1.50)
digit-span backward	COG	4.83 (1.44)	4.90 (1.01)	0.07 (1.33)
	CTRL	5.24 (1.38)	5.52 (1.21)	0.28 (1.25)
Digit-Symbol-Test	COG	50.31 (6.18)	54.41 (7.16)	4.10 (5.28)
	CTRL	53.45 (8.35)	58.59 (9.80)	5.14 (5.04)
Stroop (s)[1]	COG	39.52 (7.08)	37.69 (8.82)	-1.83 (5.64)
	CTRL	37.93 (7.43)	36.48 (9.05)	-1.64 (6.57)
Stroop difference (s)[1]	COG	17.34 (5.30)	16.38 (7.16)	-0.97 (5.74)
	CTRL	16.07 (5.01)	14.26 (6.41)	-2.34 (7.73)
**Test Battery for Attention Assessment (TAP)**				
Auditive condition (ms)[1]	COG	595.37 (100.90)	586.15 (101.02)	-9.22 (78.50)
	CTRL	574.93 (86.06)	569.59 (100.58)	-5.34 (61.59)
Visual condition (ms)[1]	COG	849.93 (119.20)	836.57 (84.56)	-13.36 (123.51)
	CTRL	828.45 (109.81)	817.38 (99.88)	-11.07 (98.85)
Total number of errors[1]	COG	2.85 (3.56)	1.65 (2.95)	-1.19 (3.25)
	CTRL	1.52 (1.79)	0.28 (0.53)	-1.24 (1.86)
**Verbal Learning and Memory Test (VLMT)**				
Total number of words	COG	43.69 (8.75)	48.03 (9.96)	4.34 (6.59)
	CTRL	53.00 (8.96)	51.24 (8.47)	-1.76 (6.37)
Immediate reproduction[1]	COG	2.24 (1.77)	1.69 (1.93)	-0.55 (2.15)
	CTRL	1.76 (1.57)	1.38 (1.47)	-0.38 (2.23)
Delayed reproduction[1]	COG	2.21 (2.23)	1.66 (1.93)	-0.55 (2.31)
	CTRL	2.00 (2.05)	1.38 (1.80)	-0.62 (2.49)
Recognition performance	COG	11.11 (3.34)	12.25 (2.91)	1.14 (3.04)
	CTRL	11.86 (3.56)	12.17 (2.89)	0.31 (2.66)
**Trail Making Test (TMT)**				
Version A (s) [1]	COG	32.86 (9.99)	28.07 (9.64)	-4.79 (9.29)
	CTRL	26.10 (7.48)	26.69 (7.92)	0.59 (6.29)
Version B (s) [1]	COG	74.28 (19.44)	65.97 (18.87)	-8.31 (17.05)
	CTRL	64.00 (18.60)	60.45 (19.98)	-3.55 (16.31)

Difference B - A (s)[1]	COG<br>CTRL	41.41 (18.15)<br>37.90 (16.21)	37.90 (14.21)<br>33.76 (17.72)	-3.52 (18.58)<br>-4.14 (16.73)
		**Cognitive Failures Questionnaire (CFQ)**		
Total score	COG<br>CTRL	59.89 (8.87)<br>61.76 (11.73)	57.48 (8.48)<br>56.83 (11.18)	-2.41 (8.03)<br>-4.93 (5.79)

Notes. COG = cognitive training group; CTRL = Control group; *N* = sample size; *M* = mean; *SD* = standard deviation; *p* = significance level. [1] Lower value represent higher performance

Supplementary Table 4

Descriptive statistics in the cognitive training group and the waiting control group at T1 and T2 in Sample 2.

**Variable**	**Group**	**T1**<br>***M (SD)***	**T2**<br>***M (SD)***	**Difference T2-T1**<br>***ΔM (SD)***
		**Attentional endurance test (d2)**		
Total number of symbols	COG<br>CTRL	418.42 (70.59)<br>440.48 (76.32)	437.46 (78.92)<br>459.65 (72.09)	19.04 (50.09)<br>19.13 (47.59)
Total Number of correct symbols	COG<br>CTRL	383.73 (67.39)<br>420.94 (75.29)	405.42 (72.83)<br>434.94 (76.99)	21.69 (36.05)<br>14.00 (53.55)
Total number of errors[1]	COG<br>CTRL	34.69 (25.51)<br>19.84 (16.72)	32.04 (33.32)<br>18.26 (15.79)	-2.65 (30.31)<br>-1.58 (14.79)
Concentartion performance	COG<br>CTRL	140.27 (33.26)<br>166.13 (36.53)	151.88 (37.08)<br>174.45 (34.61)	11.62 (22.64)<br>8.32 (19.08)
		**Performance Testing System (LPS)**		
LPS-1	COG<br>CTRL	17.54 (5.55)<br>18.71 (5.97)	17.54 (6.44)<br>21.45 (6.23)	0.00 (3.86)<br>2.74 (3.39)
LPS-3	COG<br>CTRL	22.88 (4.40)<br>27.00 (5.18)	26.08 (5.49)<br>28.87 (5.10)	3.19 (3.60)<br>1.87 (3.39)
LPS-6	COG<br>CTRL	29.50 (7.18)<br>32.61 (7.70)	33.50 (7.69)<br>32.13 (7.86)	4.00 (4.89)<br>-0.48 (9.27)
LPS-7	COG<br>CTRL	19.23 (6.33)<br>23.06 (5.68)	25.00 (6.97)<br>24.16 (8.55)	5.77 (6.38)<br>1.10 (7.28)
		**Nürnberg Age Inventory (NAI)**		
digit-span forward	COG<br>CTRL	6.23 (0.91)<br>6.26 (1.06)	6.77 (1.18)<br>6.97 (1.08)	0.54 (1.30)<br>0.71 (1.01)
digit-span backward	COG<br>CTRL	5.12 (1.03)<br>5.32 (1.35)	5.27 (1.19)<br>5.68 (1.11)	0.15 (1.29)<br>0.35 (1.14)

Digit-Symbol-Test	COG	56.35 (8.62)	56.19 (8.78)	-0.15 (6.10)
	CTRL	55.16 (10.81)	60.29 (9.71)	5.13 (5.96)
Stroop (s)[1]	COG	38.69 (8.89)	35.27 (9.15)	-3.42 (6.59)
	CTRL	35.94 (14.91)	30.00 (6.99)	-5.94 (8.93)
Stroop difference (s)[1]	COG	15.96 (7.28)	13.19 (7.65)	-2.77 (6.72)
	CTRL	14.42 (10.52)	10.84 (4.94)	-3.58 (7.39)
**Test Battery for Attention Assessment (TAP)**				
Auditive condition (ms)[1]	COG	595.37 (100.90)	586.15 (101.02)	-9.22 (78.50)
	CTRL	574.93 (86.06)	569.59 (100.58)	-5.34 (61.59)
Visual condition (ms)[1]	COG	849.93 (119.20)	836.57 (84.56)	-13.36 (123.51)
	CTRL	828.45 (109.81)	817.38 (99.88)	-11.07 (98.85)
Total number of errors[1]	COG	2.85 (3.56)	1.65 (2.95)	-1.19 (3.25)
	CTRL	1.52 (1.79)	0.28 (0.53)	-1.24 (1.86)
**Verbal Learning and Memory Test (VLMT)**				
Total number of words	COG	51.00 (6.24)	54.19 (6.54)	3.19 (7.04)
	CTRL	53.19 (9.45)	49.68 (9.41)	-3.52 (6.88)
Immediate reproduction[1]	COG	1.50 (1.24)	1.65 (2.12)	0.15 (2.05)
	CTRL	1.71 (2.21)	1.84 (1.75)	0.13 (2.53)
Delayed reproduction[1]	COG	1.58 (1.27)	1.50 (1.68)	-0.08 (1.47)
	CTRL	1.97 (2.18)	1.90 (2.09)	-0.06 (2.44)
Recognition performance	COG	12.73 (2.39)	12.92 (2.08)	0.19 (1.58)
	CTRL	12.35 (2.79)	11.55 (3.37)	-0.81 (3.04)
**Trail Making Test (TMT)**				
Version A (s) [1]	COG	30.77 (7.38)	26.23 (8.91)	-4.54 (7.52)
	CTRL	24.55 (7.08)	22.00 (7.18)	-2.55 (5.69)
Version B (s) [1]	COG	76.85 (30.37)	59.15 (19.95)	-17.69 (24.41)
	CTRL	63.45 (23.84)	53.13 (19.42)	-10.32 (15.23)
Difference B - A (s) [1]	COG	46.08 (27.94)	32.92 (16.63)	-13.15 (24.26)
	CTRL	38.90 (19.49)	31.13 (15.77)	-7.77 (14.55)
**Cognitive Failures Questionnaire (CFQ)**				
Total score	COG	53.92 (9.81)	53.74 (9.51)	-0.18 (8.13)
	CTRL	58.91 (10.45)	54.45 (9.67)	-4.46 (7.43)

Notes. COG = cognitive training group; CTRL = Control group; *N* = sample size; *M* = mean; *SD* = standard deviation; *p* = significance level. [1] Lower values represent higher performance

Differential effects of cognitive training: effects of Age, Shift work and Baseline performance

In Sample 1, the auditory task (TAP) showed an interaction Session × Group × Shift Type ($F(1, 42) = 4.81$; $p = .034$), which was due to the improvement in the COG group with night work from M = 614 ms at T1 to M = 547 ms at T2 ($F(1, 7) = 21.94$; $p = .002$), whereas the performance in the remaining groups did not change (COG without night work (M = 587 ms vs. M = 602 ms), CTRL with night work (M = 548 ms to M = 558 ms), CTRL without night work (M = 591 ms to M = 580 ms) (all F's < 1).

For the concentration performance (d2), the analysis revealed a trend toward significant interaction Session × Group × Age × Baseline Performance ($F(1, 44) = 3.34$; $p = .074$), suggesting a trend for improvement in older participants in the CTRL group with low baseline performance from 129.57 to 151.00 ($F(1, 6) = 20.61$; $p = .004$). The older participants in the COG group with low cognitive baseline performance remained unchanged in their performance (M = 120.63 to M = 119.00).

Moreover, the analyses revealed a main effect of Age for the number of errors in the visual-auditive task (TAP) ($F(1, 52) = 4.42$; $p = .04$), a main effect of Shift Type ($F(1, 52) = 10.39$; $p = .002$) as well as an interaction of both factors ($F(1, 52) = 5.83$; $p = .019$), suggesting lower performance in young participants with night work vs. without night work ($p < .001$), but no differences between shift type in older participants.

Furthermore, an interaction Age x Shift Type was found for the Stroop test ($F(1, 52) = 5.20$; $p = .027$) again with the lowest performance in young participants with night shift (M = 44.1 sec) compared to older group with night shift (M = 37.3 sec) or younger group without night shift (M = 37.3 sec). Moreover, there was an interaction for the delayed recall of words in VLMT ($F(1, 52) = 4.81$; $p = .033$) with lowest performance in younger workers with night shift compared to all other groups.

TMT-B showed a main effect of Age ($F(1, 54) = 5,08$; $p = .028$), indicating slower performance in older (M = 79.3 sec) than younger participants (M = 64.2 sec) regardless of Shift Type. The same was true for the difference TMT B-A ($F(1, 54) = 5.22$; $p = .026$): the younger participants responded faster (M = 34.1 sec) than the older participants (M = 46.3 sec).

For the digit-span backward (DS-B) the interaction Session × Group did not reach significance ($F(1, 24) = 1.02$; $p = .321$), although participants in the COG group without night work with low baseline scores showed an increase in performance from M = 3.91 to M = 4.82 ($F(1, 10) = 10.20$; $p = .01$), while participants in the CTRL group without night work with low baseline scores did not show performance change (M = 4.20 to M = 4.60). Participants in the COG group with night work and low baseline scores (M = 3.86 to M = 4.29), participants in the COG group with night work and high baseline scores (M = 6.00 to M = 6.50), the participants in the of the CTRL group with night work and high baseline values (M = 6.67 to M = 5.67), and the participants in the CTRL group with night work and low baseline values (M = 4.00 to M = 5.25) did not show statistically relevant changes.

In LPS-3, the analysis yielded an interaction Session × Group × Baseline Performance that tended to be significant ($F(1, 44) = 3.93$; $p = .054$). This could be attributed to an improvement in participants in the COG group with high baseline performance from M = 27.09 to M = 30.64 symbol

mappings (F(1, 10) = 20.12; p = .001), whereas there were no significant changes for participants in the CTRL group with high baseline cognitive scores (M = 28.36 to M = 29.07).

In Sample 2, the analysis of the visual task of the TAP yielded an interaction Session × Group × Shift Type × Baseline Performance (F(1, 42) = 8.33; p = .006), indicating faster responses of participants in the COG group with high baseline scores and night work, from M = 762 ms to M = 730 ms (F(1, 4) = 8.22; p = .046). No changes in participants in the CTRL group with night work and high baseline scores were observed.

For LPS-3, there was an interaction Session × Group × Shift Type (F(1, 42) = 5.67; p = .022): the participants in the COG group with night work showed a performance increase from M = 23.10 to M = 27.20 correct symbol assignments (F(1, 9) = 11.73; p = .008), while participants in the CTRL group with night work remained unchanged in their performance (M = 26.30 to M = 26.50).

Regarding Age and Shift Type, there was an effect of Age (F(1, 54) = 8.59; p = .005) and an interaction of Age and Shift Type in LPS-7 (F(1, 54) = 4.19; p = .045). In contrast to Sample 1, the older workers with night work showed descriptively the lowest performance compared with other groups. Also, the interaction Age x Shift Type was found for the total number of symbols in the d2 test (F(1, 54) = 4.56; p = .046) with lowest performance in older night shift workers (M = 389) and the highest performance in young night shift workers (M = 450).

Finally, a main effect of Age was found for the Stroop test (F(1, 53) = 4.85; p = .032) and TMT-B (F(1, 53) = 4.27; p = .044), suggesting slower responses in older than younger adults.

As to age groups, older participants showed a weaker baseline cognitive performance than younger participants for the domains spatial reasoning (LPS-7), interference processing (Stroop), attentional endurance (d2), shifting (TMT B and TMT B-A). Only in the error score of the test battery for attentional and executive processing (TAP) the older workers outperformed the younger ones.

Descriptive comparison between the purely cognitive training (T1 vs. T2) and the combined intervention (T2 vs. T3)

In Sample 1 the comparison of all variables in d2 - except for "sum of all errors" - showed a higher improvement in the STR/HEDE + COG group. The largest difference was shown with respect to the "concentration performance." The score in the COG group increased from M = 142.03 to M = 151.55 after training, while the STR/HEDE + COG group improved from M = 164.17 to M = 179.62. For "sum of all errors," the COG group reduced number of errors by an average of M = -3.59 errors (M = 32.86 to M = 29.28), while STR/HEDE + COG group lowered their number of errors from M = 18.48 to M = 15.59 errors after their training (M = -2.89). In LPS-6 STR/HEDE + COG group increased the number of generated words from M = 33.28 to M = 36.62 words (M = +3.34). The COG group increased performance slightly from M = 29.48 to M = 30.38 words after training (M = +0.89). In LPS-7, the COG group improved from M = 17.83 to M = 21.97 symbols on average (M = +4.14), while the STR/HEDE + COG group slightly deteriorated from M = 21.34 to M = 20.83 (M = -0.51). The same was observed regarding the "sum of reproduced words" (trial 1 to trial 5, VLMT). The COG group was able to recall more words after the intervention (M = 43.69 to M = 48.03; M = +4.34), whereas the STR/HEDE + COG group was able to recall fewer words after training (M = 53.00 to M = 51.24; M = -1.76). Daily cognitive inattentiveness, assessed by the CFQ, decreased in

the COG group from *M* = 59.89 to *M* = 57.48 (*M* = -2.41). In the STR/HEDE + COG group the mean score decreased more than twice from *M* = 61.76 to *M* = 56.83 (*M* = -4.93).

In Sample 2, the performance increase in the COG group was higher than in the STR/HEDE + COG group with respect to the variables of d2 (except "total number of symbols”). The COG group also achieved greater enhancements than the STR/HEDE + COG group for the subtests LPS-6: the performance of the STR/HEDE + COG group decreased from *M* = 32.61 words generated to *M* = 32.13 words after training (*M* = -0.48). The COG group increased performance from *M* = 29.50 to *M* = 33.50 words (*M* = +4.00). In LPS-7, the number of rotated symbols correctly recognized increased in COG group from *M* = 19.23 before training to *M* = 25 after training (*M* = +5.77). The STR/HEDE + COG group was able to match one more symbol on average after the intervention (*M* = 23.06 to *M* = 24.16). In the Digit-Symbol-Test, however, the performance of the COG group remained unchanged (*M* = 56.35 to *M* = 56.19), while the STR/HEDE + COG group improved from *M* = 55.16 to *M* = 60.29 symbol matching (*M* = +5.13). In TMT-B, the time needed for task completion in the COG group decreased from *M* = 76.85 to *M* = 59.15 seconds after the training (*M* = -17.70), while the STR/HEDE + COG improved from an average of *M* = 63.45 seconds to *M* = 53.13 seconds (*M* = -10.32). In terms of the "total error number" of the auditory and visual task (TAP), the COG group reduced their error number from *M* = 4.15 to *M* = 1.46 errors on average (*M* = -2.69). In the STR/HEDE + COG group, the number of errors remained unchanged (*M* = 2.13 to *M* = 2.00). The Cognitive Failures Questionnaire (CFQ) score remained unchanged in the COG group (*M* = 53.92 to *M* = 53.74), while in the STR/HEDE + COG, the everyday experienced cognitive failures decreased from *M* = 58.91 to *M* = 54.45 (*M* = -4.46).

References

Abel, T., Kohlmann, T. & Noack, H. (1995). Eine deutsche Übersetzung des SOC. Universität Bern: Abteilung für Gesundheitsforschung des Instituts für Sozial- und Präventivmedizin. In S. Singer & E. Brähler (Eds.), *Die „Sense of Coherence Scale”. Testhandbuch zur deutschen Version.* Göttingen: Vandenhoeck & Ruprecht.

Antonovsky, A. (1987). *Unraveling the mystery of health. How people manage stress and stay well.* San Francisco: Jossey-Bass.

Brickenkamp, R. (2002). *Test d2 – Aufmerksamkeits-Belastungs-Test* (9th. ed.). Göttingen: Hogrefe.

Broadbent, D. E., Cooper, P. F., Fitzgerald, P., & Parkes, K. R. (1982). The Cognitive Failures Questionnaire (CFQ) and its correlates. *British Journal of Clinical Psychology*, *21*(1), 1–16. https://doi.org/10.1111/j.2044-8260.1982.tb01421.x

Büssing, A. & Perrar, K. M. (1992). Die Messung von Burnout. Untersuchung einer Deutschen Fassung des Maslach Burnout Inventory (MBI-D). *Diagnostica, 38*, 328-353.

Fliege, H., Rose, M., Arck, P., Levenstein, S. & Klapp, B. F. (2001). Validierung des „Perceived Stress Questionnaire“ (PSQ) an einer deutschen Stichprobe. *Diagnostica, 47*(3), 142-152.

Franke, A., & Witte, M. (2009). *Das HEDE-Training®: Manual zur Gesundheitsförderung auf Basis der Salutogenese*. Hogrefe AG.

Gajewski, P. D., & Falkenstein, M. (2012). Training-Induced Improvement of Response Selection and Error Detection in Aging Assessed by Task Switching: Effects of Cognitive, Physical, and Relaxation Training. *Frontiers in Human Neuroscience*, *6*. https://doi.org/10.3389/fnhum.2012.00130

Helmstaedter, C., Lendt, M. & Lux, S. (2001). *Verbaler Lern- und Merkfähigkeitstest*. Weinheim: Beltz.

Ilmarinen, J. (2009). Work ability—a comprehensive concept for occupational health research and prevention. *Scandinavian Journal of Work, Environment &Amp; Health*, *35*(1), 1–5. https://doi.org/10.5271/sjweh.1304

Ilmarinen. (2019). From Work Ability Research to Implementation. *International Journal of Environmental Research and Public Health*, *16*(16), 2882. https://doi.org/10.3390/ijerph16162882

Goldberg, D., Gater, R., Sartorius, N., Ustun, T. B., Piccinelli, M., Gureje, O. & Rutter, C. (1997). The validity of two versions of the GHQ in the WHO study of mental illness in general health care. *Psychological medicine: a journal for research in psychiatry and the allied science,* 27(1), 191-197.

Goldberg, D. & Williams, P. (1991). *A users guide to General Health Questionnaire*. Windsor, UK: NFER-Nelson.

Horn, W. (1983). *Leistungsprüfsystem. Handanweisung* (2nd ed.). Göttingen: Hogrefe.

Jacobson, E. (2006). *Entspannung als Therapie: Progressive Relaxation in Theorie und Praxis*. Klett-Cotta.

Karbach, J., & Kray, J. (2009). How useful is executive control training? Age differences in near and far transfer of task-switching training. *Developmental Science*, *12*(6), 978–990. https://doi.org/10.1111/j.1467-7687.2009.00846.x

Lehrl, S., Lehrl, M. & Weickmann, E. (1994). *MAT Gehirnjogging, Einführung in das Mentale Aktivierungstraining*. Ebersberg: Vless Verlag.

Levenstein, S., Prantera, C., Varvo, V., Scribano, M., Berto, E., Luzi, C., & Andreoli, A. (1993). Development of the perceived stress questionnaire: A new tool for psychosomatic research. *Journal of Psychosomatic Research*, *37*(1), 19–32. https://doi.org/10.1016/0022-3999(93)90120-5

Lüthi, M. (2009). Trail Making Test (TMT). In D. Schellig, R. Drechsler, D. Heinemann & W. Sturm (Eds.), *Handbuch neuropsychologischer Testverfahren. Aufmerksamkeit, Gedächtnis und exekutive Funktionen* (pp. 826-833). Göttingen: Hogrefe.

Maslach, C. & Jackson, S. E. (1981). The measurement of experienced burnout. *Journal of Occupational Behavior, 2*, 99-113.

Maslach, C. & Jackson, S. E. (1986). *Maslach Burnout Inventory (2. ed.)*. Palo Alto, CA: Consulting Psychologists Press.

Maslach, C., Jackson, S. E. & Leiter, M. P. (1996). *MBI: The Maslach Burnout Inventory: Manual research edition.* Palo Alto, CA: Consulting Psychologists Press.

Mittelmark, M. B., Bauer, G. F., Vaandrager, L., Pelikan, J. M., Sagy, S., Eriksson, M., Lindström, B., & Magistretti, M. C. (2021). *The Handbook of Salutogenesis* (2nd ed. 2022). Springer.

Oswald, W. D. & Fleischmann, U. M. (1999). *Nürnberger-Alters-Inventar* (4th ed.). Göttingen: Hogrefe.

Reitan, R. M. (1992). *Trail Making Test.* Tucson, A. Z.: Reitan neuropsychological Laboratory.

Reynolds C. R. (2002) Comprehensive Trail Making Test (CTMT): examiner's manual, Austin, TX: PRO-ED.

Sattler-Rommel, T. (2005). Mentales AktivierungsTraining (MAT) - Ein Weg kognitive Ressourcen zu erhalten. *Naturheilpraxis, 7*.

Schaarschmidt, U. & Fischer, A. W. (2008). *Arbeitsbezogenes Verhaltens- und Erlebensmuster (AVEM-44)*. Manual. London: Pearson.

Schaefer, S. & Bäckman, L. (2007). Normales und pathologisches Altern. In J. Brandtstädter & U. Lindenberger (Eds.), *Entwicklungspsychologie der Lebensspanne. Ein Lehrbuch* (pp. 245-269). Stuttgart: Kohlhammer.

Schmidt, M. (2016). *Rey Auditory Verbal Learning Test RAVLT: A Handbook*. Western Psychological Services.

Schumacher, J., Gunzelmann, T. & Brähler, E. (2000a). Deutsche Normierung der Sense of Coherence Scale von Antonovsky. *Diagnostica, 46*, 208-213.

Schumacher, J., Wilz, G., Gunzelmann, T. & Brähler, E. (2000b). Die Sense of Coherence Scale von Antonovsky - Teststatische Überprüfung in einer repräsentativen Bevölkerungsstichprobe und Konstruktion einer Kurzskala. *Psychotherapie, Psychosomatik, Medizinische Psychologie, 50*, 422-428.

Stroop, J. R. (1935). Studies of interference in serial verbal reactions. *Journal of Experimental Psychology*, *18*(6), 643–662. https://doi.org/10.1037/h0054651

Westerberg, H., Jacobaeus, H., Hirvikoski, T., Clevberger, P., Östensson, M. L., Bartfai, A., & Klingberg, T. (2007). Computerized working memory training after stroke–A pilot study. *Brain Injury*, *21*(1), 21–29. https://doi.org/10.1080/02699050601148726

Zimmermann, P. & Fimm, B. (2002). *Testbatterie zur Aufmerksamkeitsprüfung* (V 2.1). Herzogenrath: Psytest.
